# Supplementary material for: Protocol for the Pathways Study: a realist evaluation of staff social ties and communication in the delivery of neonatal care in Kenya
Source: BMJ Open. 2023 Mar 13;13(3):e066150. doi: 10.1136/bmjopen-2022-066150 (PMC10016238; doi:10.1136/bmjopen-2022-066150)
Supplement: Supplementary data [file bmjopen-2022-066150supp001.pdf]

Appendix A: Pathways Study: Non-participant observation prompt list

Before you start:

Introduce yourself to the staff you will be observing, explain the study, and obtain verbal consent (**record verbal consent on the relevant form**).

Note down:

- Date, time, duration, and location of non-participant observation
- Which cadres of staff/students you observe
- Numbers of staff you observe

Level A: Administration, Leadership and Management

1. **Where is the Administration block located in relation to the health care service delivery units?** Which offices and posts are located in the administration block?
2. **Is there a notice board?** What is posted on the notices?
3. **Is there a service charter?** What is contained on the charter? Where is it displayed?
4. **Is there a company/institutional mission, vision, values displayed?** Describe this.
5. **Where is the CEO's office OR medical superintendent's office in relation to other offices?** What is contained in the office: Furniture, artefacts, symbols, wall hangings?
6. **What is contained in the head of nursing's office?** Furniture, artefacts, symbols, wall hangings?
7. **What is the nature of dressing for the hospital top management?** What is the colour? Do they have the facility logo?
8. **What is the nature of dressing for hospital staff?** Does it differ from that of top management? Do they have facility logo? Are they uniforms? Are there dressings with no logos or with any other logos?
9. **How is the parking organised?** Management versus staff
10. **Which meetings were observed?** What was the purpose of the meeting? Was the meeting by formal invitation (any evidence to this?) Where were they held? Who was chair? How are people seated? Who spoke to whom? Any commonly used words/phrases? Any common verbal and non-verbal cues? **Are there any meetings before/after the main meeting?** How are the members constituted? How are they conducted? How long do they last? What happens thereafter?

Level B: Units/Wards

1. **Who are staff generally communicating with?** Within their own cadre/seniority level? Between different cadres/seniority levels?
2. **Do they communicate with different colleagues depending on what they are communicating about?** Which colleagues to staff appear to communicate freely with? Are there any barriers between certain staff? What is the style of communication of different staff?
3. **Do all staff appear to be equally free to communicate with their colleagues?** If not, who is not able to?
4. **What appear to be the norms of staff behaviour in the location you are observing?** Do some cadres of staff appear to have different behavioural norms to others?
5. **Do staff appear to follow advice of their colleagues in their work/decision-making?** Can you describe?
6. **Which staff seem to be more influential in the location you are observing?** What made you form this assessment/why?
7. **Which staff seem less influential in the location you are observing?** What made you form this assessment/why?
8. **Can you notice a hierarchy in the location you are observing?** Please describe.
9. **Do staff use any other information sources to undertake their work?** Books, protocols, etc?
10. **Can you observe any mentoring/supervising/supporting relationships or behaviours between staff?** How formal/informal is it? How is the relationship between the mentor and mentee(s)?
11. **Any unit-level meetings observed?** Describe Type, purpose of the meeting, where the meeting is held, who attended the meeting, who chairs the meeting, how are people seated? Who speaks to who? Any words or phrases that are commonly used? Any common verbal and non-verbal cues? **Are there any meetings before/after the main meeting?** How long do these last? How are the membership informally constituted? How are they are conducted? What happens thereafter?

Appendix B: Pathways Study - In-Depth Interview Guide

(Informed consent must be provided by the participant before commencing the interview.) *My name is X and I am a researcher working on the Pathways Study. I am interested in learning more about how nursing graduates (staff) who work in neonatal care utilise knowledge and skills learnt in nursing programmes, and how this is shaped their relationships at work and their peers and other clinical staff. I would like to ask you a few questions.*
Participant Study ID number:

<u>PART A – for all participants</u>

1. **How do you decide/choose who to ask for help or information at work?** Can you give me an example? Why did you choose this person? What was it about them/you/the situation, which made you ask them? Does choice of colleague depend on the type of help or information you need? Which colleagues do you need to speak to on a day-to-day basis to undertake your role effectively? Why?
2. **Does discussion with a colleague at work change what you do/ your behaviour/ your decision making?** Can you give me an example?
3. **Whose opinion(s) do you value most at work?** Why? Anything about them/you/the situation?
4. **Are there any colleagues that you find difficult to speak to (ask for help from) at work?** Why do you think you find it difficult to speak to (ask for help from) them? Anything about them/you/the situation? Are there any situations when you would speak to (ask for help from) them? Do you feel comfortable approaching senior (or junior) colleagues for help? Do you feel comfortable approaching colleagues from different professions for help?
5. **How do you select with whom to share your helpful information or knowledge (i.e. information that you have)?** What kinds of information or knowledge do you share?
6. **Are there any colleagues with whom you find it difficult to share your helpful information or knowledge?**
7. **Are there situations where you have to ask for information or help but you cannot get it?** Can you give me an example: situation/type of help/from who? What did you do about it: yourself/situation/the other person(s)? How does this make you feel?
8. **Do you feel staff members share information and knowledge freely at your hospital/unit?** Can you give me an example? What type of information, knowledge or help would people be commonly seek from others, or share with others on a day-to-day basis? Why do you think this is so?
9. **What would help you better communicate with your colleagues at work?** Would this improve patient care? How? Why?
10. **Can you always access the information you need in the workplace?** Do you ever feel like you are missing out on information at work? Do you try to access the information in any other ways? For example: clinical guidelines, protocols, hospital policies, key communication messages from management to staff, information specific to the professional cadre, books, phone, WhatsApp groups, any other information?
11. **Do you use any other information sources/resources/courses/CPD to do your job?** Can you give an example? How and when do you access this information?
12. **How could access to essential and reliable information be improved in the unit/hospital?** Would this improve patient care? How? Why?
13. **Do you feel that you can always say and do what you think is best, in the workplace?** Why/why not? Please give me an example. Do you feel like you do things differently or have a different outlook/attitude to some of your colleagues? How does this make you feel? Please give me an example. How does this make your colleagues feel? Does this impact on your patient care?
14. **What are your experiences of supervision/support/mentoring at work?**
15. **What (or who) do you feel has influenced you the most in the way you work?** Can you describe this type of influence detail? How has this impacted your individual perception about your work? How has this impacted on how you provide care to your patients? How does this make you and colleagues feel?
16. **Do you feel there is anyone at work who influences the behaviour of your whole peer group?** Who? Why do you think this is? Do you think you contribute to this person influencing your peer group? How?
17. **Are there individuals you prefer to be on duty with?** Who? What is it about them that makes you want to only work with? What is it about you that makes you wish to work with them?
18. **Are there individuals you would prefer not to be on duty with?** Who? What is it about them that makes you wish not to work with them? What is it about you that makes you wish not to work with them?
19. **What do you do if you receive contradictory advice at work?** Where does the contradictory advice come from? Talk me through your thought processes in how you decide what you will do.
20. **What are the hospital's 'values'?** How are these expressed to you? Do these 'values' change what you do at work?

21. **Can you describe what is known and said about this hospital by the general public?** Do you think this perception is also shared by some (or most) staff members? What do you think has contributed to this? How has this influenced the way you and your colleagues provide care to your patients?

For non-nurse staff and students, interview ends here.

PART B: additional questions for nurse managers, all nurses, plus student nurses

20. **What is your experience with new graduate nurses in providing care to neonates?** What makes you say so?
21. **Is there supervision/support/mentorship at workplace?** To whom is it targeted? Who implements it? When does this happen? What has been your experiences of supervision/support/mentoring at work? Can you give me an example? (nurses and student nurses)
22. **Does the workplace plan and implement off-the job formal training (including both short and long training opportunities) aimed at improving patient care at work?** Can you give an example? **If you have attended any of these trainings, c**an you comment on your experience? Do you feel you have utilised what you learned? What makes you say so? Are there any specific workplace factors that contributed to this? What are they? (nurses)
23. **What stands out as a factor(s) that determines how nurses are likely (or not) going to utilise that they have learned at their workplace?** Tell me about institutional leadership factors, mentorship factors, other institutional initiatives (which, for who, how, when)? (nurses and student nurses)
24. **Tell me about individual nurse-specific factors that you have observed that determine how likely nurses are to use what they have learned in their workplace** (looking for things like effective communication, teamwork self-awareness, decision making etc----human factors)? (nurses and student nurses)
25. **Did you feel you are confident in communicating with rude, angry or disrespectful patients/colleagues?** Why do you say so? Can you give me an example?
26. **Are there emotionally challenging situations that you find handling at workplace?** Give me an example? Why do you say so? How does this impact on the care you give to your patients?
27. **Do you think there are factors about your other professional colleagues (doctors, nutritionists, laboratory personnel etc) that contribute to how nurses utilize knowledge and skills and competencies acquired during their training?** What are these factors? Why do you say so? (nurses and student nurses)
28. **Do you think the nursing school training theoretical work (including in clinical skills labs) is aligned to what is expected at the workplace?** Can you comment on this as a student nurse, a newly qualified nurse and as an experienced manager/leader?

Appendix C: Pathways Study – Social Networks Questionnaire

Informed consent must be provided by the participant before commencing the SNA questionnaire interview.

My name is X and I am a researcher working on the Pathways Study. We are interested in learning more about how staff/students who work in neonatal care communicate with one another, and how this might influence what they do at work. I would like to ask you a few questions about your own experiences of communicating with your colleagues.

Demographics

- Study ID number
- Job title/cadre/student status (include qualification and level of seniority)
- Unit (maternity, postnatal, neonatal, paediatric)
- Year of birth (or age in years)
- Gender
- Highest qualification (and year)
- Specialist certificates
- Training courses/workshops attended (in the last 5 years)
- Involvement in research
- Academic/teaching/faculty position
- Nomination as a clinical mentor/champion/coach/preceptor
- Length of time working on ward
- Employment elsewhere

Scenarios (will be piloted and adapted at start of study)

1. You are not sure of the dose of medication to give to a neonate.
   List #1: Please indicate if you would speak to/seek advice from anyone

2. A neonate has an infection and you are unsure of the treatment plan.
   List #1: Please indicate if you would speak to/seek advice from anyone

3. You need help to perform a routine procedure on a stable neonate.
   List #1: Please indicate if you would speak to/seek advice from anyone

4. You have a patient on the ward with clinical orders that you are sure do not comply with clinical practice guidelines.
   List #1: Please indicate if you would speak to/seek advice from anyone

5. You have a patient on the ward who you think needs a certain specific care, but it is not available.
   List #1: Please indicate if you would speak to/seek advice from anyone

6. You notice that a neonate is looking very sick.
   List #1: Please indicate if you would speak to/seek advice from anyone

7. You have had a very busy shift and two neonates died today. You are feeling tired and upset.
   List #1: Please indicate if you would speak to/seek advice from anyone

8. The unit has no water.
   List #1: Please indicate if you would speak to/seek advice from anyone

9. A colleague has been rude to you.
   List #1: Please indicate if you would speak to/seek advice from anyone

10. You are very busy and some of your colleagues have finished their tasks.
    List #1: Please indicate which colleagues would likely offer to help you with your own tasks

11. A mother has refused a procedure for her baby.
    List #1: Please indicate if you would speak to/seek advice from anyone

12. A baby has died on the unit and you are uncomfortable breaking the bad news to her.

List #1: Please indicate if you would speak to/seek advice from anyone
List #1 = Roster method – a list of staff working in or supporting the unit

Appendix D1: Pathways Study – Key informant Interview

for Hospital Nursing Team Leader/Manager (one per hospital/unit)

(Informed consent must be provided by the participant before commencing the interview.) *My name is X and I am a researcher working on the Pathways Study. I am interested in learning more about how nursing graduates (staff) who work in neonatal care utilise knowledge and skills learnt in nursing programmes, and how this is shaped their relationships at work and their peers and other clinical staff. I would like to ask you a few questions.*

Participant study ID number:

Interview schedule: (Schedule will be piloted and adapted at start of study. Questions will also be adapted for interviewing researchers, as per protocol)

1. **Can you describe your role in unit** (Shift leader, unit manager etc).
2. **Does your unit/hospital have any job/role descriptions for nurses working on the unit?** Please can I see a copy?
3. **Does your unit/hospital have any Continuing Professional Development policies?** Please can I see a copy? Does the hospital have a library? Any annual CPD requirements for staff? Appraisals? Etc.
4. **Does your unit/hospital have any regular clinical meetings?** Who attends? What is the format? How frequently? Etc.
5. **Are any on-the-job training schemes going on in your unit/hospital for nurses?** Courses? Mentoring? Etc.
6. **What is your total nursing staff?** What is your current patient: nurse ratio? What is your expected ration?
7. **Do you have a nursing staff rota for each shift**? Can I have a copy of the last week and this week?
8. **How many nurses are you likely to be having on each shift?** AM, PM, Night, Weekends? What is the typical composition of this rota? Why is that so? Is this defined by the institution? Any other factors (e.g NCK etc?) (Nurses and student nurses)

Appendix D2: Pathways Study – Key informant Interview

for Nurse educators with administration and programme management role (one per teaching institution)

(Informed consent must be provided by the participant before commencing the interview.) *My name is X and I am a researcher working on the Pathways Study. I am interested in learning more about how nursing graduates (staff) who work in neonatal care utilise knowledge and skills learnt in nursing programmes, and how this is shaped their relationships at work and their peers and other clinical staff. I would like to ask you a few questions.*

Participant study ID number:

Interview schedule: (Schedule will be piloted and adapted at start of study. Questions will also be adapted for interviewing researchers, as per protocol)

9. **What is your role in the college** (Lecturer/tutor, programme manager, head of department etc).
10. **How many nursing programmes do you have in the school?** What is the level of training for each? What is the course duration of each?
11. **Do you have a curriculum for your nursing programmes?** Can I have a copy please? **What is your curricular review/revision policy/plan?** Can I have a look at it please?
12. **Are the nursing programmes all accredited?** Who is the accreditor? Can I see evidence of accreditation please?
13. **What is the entry criteria for each?** How do you determine the entry criteria? Do you have evidence for this?
14. **What is the admission capacity for each programme?** How is this determined?
15. **How many nurses from the programmes graduate annually?** Do you have any dropouts?
16. **Where do your nursing students undertake their clinical practicum?** Do you have any formal agreements with these institutions? Can I see the evidence please?
17. **Is there any mutual working relationship with facilities that provide student practicum placements in the area of nursing staff development trainings (workshops, symposiums, library, clinical consultancy)?** If you have evidence for this, can I see it please?
18. **What is your total academic staff (includes clinical laboratory and clinical instructors)?** What is your current Student: Faculty ratio? What is your expected ratio?

Appendix E1 - Pathways Study - FGD: Nursing educators (Teaching faculty involved in the curriculum implementation of nurse trainings for both basic and specialist neonatal and/or paediatric nurses, 5-9 members per FGD)

(Informed consent must be provided by each of the participant before commencing the discussion.) *My name is X and I am a researcher working on the Pathways Study. I am interested in learning more about how basic and specialist nursing graduates (staff) who work in neonatal care units are prepared for their workplace and how this preparation influences how they utilise knowledge and skills learnt in their nursing training. I would like us to have a discussion about your own experiences.*

1. Welcome the participants, do a formal introduction and allocate **Participant study ID numbers:**
2. Explain the process and lay ground rules
3. Explain the need to record
4. Gain verbal consent for participation and recording

Main question	Additional questions
What is your experience of how well Kenya's nurse training programmes prepare nurses to work in neonatal care units?	• Start off with basic programmes<br>• Then ask about Specialist programmes<br><br>Ask Why they say so? Give examples
In your experience as educators, what has worked to adequately prepare any nurse, basic or specialist, to be able to easily use what they have learned in college at their workplaces?	Probe on people-related factors<br>Probe on leadership related factors<br>Probe on assertiveness, self-awareness and decision-making factors
In your experience as educators, what has hindered preparing any nurse, basic or specialist, to be able to easily use what they have learned in college at their workplaces?	Probe on people-related factors<br>Probe on leadership related factors<br>Probe on assertiveness, self-awareness and decision-making factors
Do you think the theoretical work (including in clinical skills labs) is aligned to what is expected at the workplace for your future graduate nurses?	Probe on how this is scheduled, delivered and perceived by both teachers and learners
Do you think the communication skills and emotional competence skills taught at the nursing school is aligned to the challenges nurse graduates face at the workplace?	Probe on how these skills are taught and perceived by both teachers and learners
In your experience as educators, what things/factors about nursing students themselves have helped them, basic or specialist, to be able to easily use what they have learned in college at their workplaces?	Probe on people-related factors<br>Probe on leadership related factors<br>Probe on assertiveness, self-awareness and decision-making factors
In your experience as educators, what things/factors about nursing students themselves have hindered them, basic or specialist, to be able to easily use what they have learned in college at their workplaces?	Probe on people-related factors<br>Probe on leadership related factors<br>Probe on assertiveness, self-awareness and decision-making factors
In your experience as educators, what things/factors about hospital staff as clinical preceptors/clinical mentors do you think have helped nurses, basic	Probe on people-related factors<br>Probe on leadership related factors

or specialist, to be able to easily use what they have learned in college at their workplaces?	Probe on assertiveness, self-awareness and decision-making factors
In your experience as educators, what things/factors about hospital staff as clinical preceptors/clinical mentors have hindered nurses, basic or specialist, to be able to easily use what they have learned in college at their workplaces?	Probe on people-related factors<br>Probe on leadership related factors<br>Probe on assertiveness, self-awareness and decision-making factors
Do you think there are factors about your other professional colleagues (doctors, nutritionists, laboratory personnel etc) that contribute to how nurses utilize knowledge and skills and competencies acquired during training?	Probe to identify these factors (probe on teamwork, team leadership, decision making and other human factor development through collaborative social learning)<br><br>Why do you say so?
What has been the role of the teaching institution (any initiatives) in responding to the challenges you have mentioned above: challenges at<br>• Teaching faculty level<br>• Students' level<br>• Hospital staff level	Probe further on these initiatives, what, when, how, for whom?
Are you involved in these initiatives?	Probe further on this: What, as who, when, how, for whom?
Do you have any recommendations going forward?	
Is there anything else from anyone before we close?	
Thank the participants for their time and contributions	

Appendix E2 - Pathways Study - FGDs: 1) Nursing graduates **(Basic Diploma and Degree General Nursing graduates)** working in various units and providing caring for newborn babies, and **2) Specialist Nursing graduates (Specialist Neonatal and/or Paediatric Nurses and/or maternal-neonatal nurses)** working in various units and providing caring for newborn babies (5-9 members per FGD).

(Informed consent must be provided by each of the participant before commencing the discussion.) *My name is X and I am a researcher working on the Pathways Study. I am interested in learning more about how nursing graduates (staff) who work in neonatal care utilise knowledge and skills learnt in nursing programmes or other neonatal short CPD programmes. I would like us to have a discussion about your own experiences.*

5. Welcome the participants, do a formal introduction and allocate **Participant study ID numbers:**
6. Explain the process and lay ground rules
7. Explain the need to record
8. Gain verbal consent for participation and recording

Main question	Additional questions
Can you describe to me the nature of work you are involved in in this unit/department?	Are there any challenges you have experienced in undertaking your role? Give examples and why?
For Basic Diploma/Degree General Nurses: How would you describe your basic training in preparing you to work in neonatal care units?<br><br>*For Specialist Neonatal and/or Paediatric Nurses and/or Maternal-neonatal Nurses:* How would you describe your specialist training in preparing you to work in neonatal care units? How does this compare with your basic nursing training?	Why do you say so? Give examples<br><br>Probe on preparedness on communication skills and handling emotions (ability to communicate well with patients, colleagues, and supervisors. Handling angry parents, breaking bad news & handling rude/ disrespectful colleagues)<br>Recognizing and managing own stress
Have you undertaken any neonatal-training relevant short course/training? Describe it: what, when, content, how and relevance	How does it compare to your basic nursing training? Why do you say so? Give examples.
What things/factors about yourself have helped you use what you learned in college and/or in short courses?	Probe on people-related factors<br>Probe on leadership related factors<br>Probe on assertiveness, self-awareness and decision-making factors
What things/factors about working with others have helped you use what you learned in college and/or in short trainings?	Probe on people-related factors<br>Probe on leadership related factors<br>Probe on assertiveness, self-awareness and decision-making factors
What things/factors about yourself have not helped you use what you learned in college and/or short trainings?	Give examples. Probe as above. Are these factors shared by others? What do the rest of us think?

What things/factors about working with others have hindered your ability to use what you learned in college and/or short trainings?	Give examples. Probe as above. Are these factors shared by others? What do the rest of us think?
Do you think your colleagues elsewhere share your feelings? (ask about Private hospitals versus public hospitals)	Why do you think this is so?
What is your opinion about practice improvement initiatives such as?<br>1. CPD courses (give examples)<br>2. Workplace mentorship<br>3. Using clinical practice guidelines<br>4. Staff induction trainings<br>5. Staff appraisal and performance management	Probe on How it is done, Why it is done, for Whom it is done, When it is done
If you are involved in any of the mentioned practice improvement initiatives above (need to say which one), share your experiences.	Ask: whether the experience is as a recipient or as a provider. Ask them to describe: How, When and Why.
Do you have any recommendations about this?	Ask about:<br>1. Recommendations for nurses as practitioners<br>2. Recommendations about other clinical colleagues<br>3. Recommendations for hospital managers<br>4. Recommendations for Nursing schools<br>5. Recommendations for the regulator
Is there anything else from anyone before we close?	
Thank the participants for their time and contributions	

Appendix F - Pathways Study – Stakeholder workshop event schedule (10-15 members)

Event programme/ timetable (if to be held online instead of in-person, the programme will be adapted accordingly)

Time	Activity	Description
10am	Consent<br>Registration and refreshments	Invited stakeholders will be asked to give consent before participating in the event. They will have previously received a participant information sheet by email and will be given the opportunity to ask any questions in person, before completing the consent form.<br>Reimbursements will be given to participants.
10.30am	Welcome	Participants will be welcomed to the event by the research team.
10.45am	Introduction to study	Researchers will present the background of the study, including findings of a realist synthesis, and work conducted on the neonatal unit in Kenya.
11.15am	Group brainstorming session – how do social ties influence quality of patient care?	Participants will be asked to discuss their initial thoughts on the information/issues in the presentation and how this compares to personal experiences, in small groups. Groups will be provided with paper, sticky notes, and flip chart.
11.45am	Feedback to whole group	Each small group will be encouraged to share their thoughts and take any questions.
12.15pm	Lunch	
1pm	Presentation of findings from interviews, SNA and non-participant observation	Researchers will present the remaining findings of the work conducted on the neonatal unit in Kenya to the stakeholders. This will include a series of summary slides and handouts which the participants can use in the activity.
1.30pm	Group activity – what do we think of the findings? how should these findings be used?	Participants will be asked to discuss the findings and put down their thoughts on paper, to be able to present back to the whole group.
2pm	Feedback to whole group	Each small group will be encouraged to share their thoughts and take any questions.
2.30pm	Tea	
3pm	Group discussion – what should our recommendations be?	During the tea break, the researchers will have collated the main points from the previous discussions and summarised into themes. These themes will be used as prompts for discussion.<br>The session will be flexible, adapting based on the dynamics of previous sessions but is likely to involved facilitated group work, facilitated group discussions, and ranking exercises or similar.
4.30pm	Summary and close	Stakeholders will be thanked for their participation and given information about how findings will be fed back to them and others.
