# Supplementary material for: Protocol for the Pathways Study: a realist evaluation of staff social ties and communication in the delivery of neonatal care in Kenya
Source: BMJ Open. 2023 Mar 13;13(3):e066150. doi: 10.1136/bmjopen-2022-066150 (PMC10016238; doi:10.1136/bmjopen-2022-066150)
Supplement: Author's manuscript [file bmjopen-2022-066150.draft_revisions.pdf]

BMJ Open

BMJ Open is committed to open peer review. As part of this commitment we make the peer review history of every article we publish publicly available.

When an article is published we post the peer reviewers' comments and the authors' responses online. We also post the versions of the paper that were used during peer review. These are the versions that the peer review comments apply to.

The versions of the paper that follow are the versions that were submitted during the peer review process. They are not the versions of record or the final published versions. They should not be cited or distributed as the published version of this manuscript.

BMJ Open is an open access journal and the full, final, typeset and author-corrected version of record of the manuscript is available on our site with no access controls, subscription charges or pay-per-view fees (http://bmjopen.bmj.com).

If you have any questions on BMJ Open's open peer review process please email info.bmjopen@bmj.com

BMJ Open

Protocol for the Pathways Study: a realist evaluation of staff social ties and communication in the delivery of neonatal care in Kenya

Journal:	*BMJ Open*
Manuscript ID	bmjopen-2022-066150
Article Type:	Protocol
Date Submitted by the Author:	22-Jul-2022

Complete List of Authors:	Wanyama, Conrad; KEMRI-Wellcome Trust Research Programme<br>Blacklock, Claire; University of Oxford, Nuffield Department of Medicine<br>Jepkosgei, Juliet; KEMRI-Wellcome Trust Research Programme<br>English, Mike; Univerity of Oxford Nuffield Department of Medicine, Nuffield Department of Medicine and Department of Paediatrics; KEMRI-Wellcome Trust Research Programme Nairobi,<br>Hinton, Lisa; University of Cambridge, The Healthcare Improvement Studies Institute<br>McKnight, Jacob ; Univerity of Oxford Nuffield Department of Medicine, Tropical Medicine; University of Oxford Nuffield Department of Clinical Medicine<br>Molyneux, Sassy ; University of Oxford, Nuffield Department of Medicine; KEMRI-Wellcome Trust Research Programme, Centre for Geographic Medicine Research-Coast<br>Boga, Mwanamvua ; KEMRI-Wellcome Trust Research Programme, Centre for Geographic Medicine Research-Coast<br>Musitia, Peris; KEMRI-Wellcome Trust Research Programme Nairobi, Health Services Unit; KEMRI-Wellcome Trust Research Programme Nairobi, Health Services Unit<br>Wong, Geoff; Oxford University, Nuffield Department of Primary Care Health Sciences

Keywords:	Health policy < HEALTH SERVICES ADMINISTRATION & MANAGEMENT, Quality in health care < HEALTH SERVICES ADMINISTRATION & MANAGEMENT, NEONATOLOGY

SCHOLARONE™
Manuscripts

Protocol for the Pathways Study: a realist evaluation of staff social ties and communication in the delivery of neonatal care in Kenya

Wanyama C*[1], Blacklock C*[2], Jepkosgei J[1], English M[1,2], Hinton L[3], McKnight J[2], Molyneux S[1,2], Boga M[1], Musitia P[1], Wong G[4].

*Joint first authors

[1]Health Services Unit, KEMRI-Wellcome Trust Research Programme, Nairobi, Kenya

[2]Nuffield Department of Medicine, University of Oxford, UK

[3]The Healthcare Improvement Studies Institute, University of Cambridge, UK

[4]Nuffield Department of Primary Care Health Sciences, University of Oxford, UK

Corresponding author:

Conrad Wanyama[1],

KEMRI-Wellcome Trust Research Programme

P.O Box 43640-00100

Nairobi, Kenya

cwanyama@kemri-wellcome.org

For peer review only

Abstract

Title: A realist evaluation of staff social ties and communication in the delivery of neonatal care in Kenya

Introduction: Initiatives to improve the quality of neonatal care in low- and middle-income countries are vital to meet the Sustainable Development Goal 3 of reducing up to 12 neonatal deaths per 1,000 by the year 2030. These initiatives have included enhancing human resources for health (HRH). However, the informal social ties that health workers form with colleagues and how this influences the application of learned knowledge and skills and individual and group behaviours and norms in the workplace, have remained neglected in health systems research.

Aims: This study seeks to better understand the relational components among health workers in Kenyan neonatal care areas, and how such understanding might inform the design and implementation of quality improvement interventions targeting health workers' behaviours.

Methods: We will collect data in two phases. In Phase One we will conduct non-participant observation of hospital staff during patient care and hospital meetings, a social network questionnaire with staff, in-depth interviews, key informant interviews, and focus group discussions at 2 large public hospitals in Kenya. Data will be collected purposively and analysed using realist evaluation, interim analyses including thematic analysis of qualitative data and quantitative analysis of social network metrics. In Phase Two, a stakeholder workshop will be held to discuss and refine phase one findings.

Results and Conclusion: Study findings will help refine an evolving programme theory with recommendations used to develop theory-informed interventions targeted at enhancing quality improvement efforts in Kenyan hospitals.

Ethics and dissemination: The study has been approved by Kenya Medical Research Institute (KEMRI/SERU/CGMR-C/241/4374) and Oxford Tropical Research Ethics Committee

For peer review only

For peer review only - http://bmjopen.bmj.com/site/about/guidelines.xhtml

(OxTREC 519-22). Research findings will be shared with the sites, and, disseminated in seminars, conferences, and, published in open-access scientific journals.

For peer review only

For peer review only - http://bmjopen.bmj.com/site/about/guidelines.xhtml

Strengths and limitations of this study

Strengths

- Realist evaluation will enable the development of programme theory, which will be useful for informing the practical design and implementation of quality improvement interventions in neonatal units.
- The Pathways Study is the first to use social network analysis to explore the influence of staff social ties on the delivery and quality of neonatal care in Kenya.

Limitations

- Relational ties and other aspects of health systems 'software' are notoriously difficult to capture and measure in health systems research.
- The Pathways Study uses a mixed methods approach to collect diverse data, but it is possible that some relevant data may still not be captured. The research team will mitigate this risk by using an iterative and exploratory approach to data collection and analysis, seeking triangulation of emergent findings, promoting reflexivity of the research team, and sense-checking emergent findings with relevant stakeholders, whilst at the same time comparing with substantive sociological theory.

For peer review only

Introduction

Despite significant reductions in mortality rates across other age groups among children under-5 in recent years, the neonatal mortality rate in Kenya has remained relatively unchanged.(1,2) In response to this disparity, efforts are ongoing to improve the quality of neonatal care, the majority of which is delivered from public health facilities and mostly by nurses.(3) Such improvement efforts have included the recent introduction of new technologies in selected neonatal units, establishing routine neonatal data collection and feedback systems, and ongoing investment in the neonatal workforce through training and workplace support.(4,5)

However, a more detailed understanding of tacit human factors, or health systems 'software',(6) that also influence the quality of neonatal care delivered in these hospitals is now needed, to inform better focused design and implementation of improvement efforts (e.g. the relationships between staff, values, norms and the social and cognitive skills that complement clinical technical skills).(7) Indeed, such 'software' factors can profoundly influence acquisition of new knowledge and behaviours and norms at individual and group level,(8–11) and thus are determinants of quality care.(12,13) Moreover, individual health worker performance is shaped not only by technical clinical competencies and experiences, but also by social and cognitive skills (cognitive, social and personal resource skills such as communication, team work, leadership, situational awareness, assertiveness and decision making, coping with stress and managing fatigue etc).(14) Relational ties among the workforce and other 'software' aspects matter greatly in the delivery of quality neonatal care, for example care pathways, clinical guidelines, knowledge, and other competencies, including those that are transferred from an educational setting such as those gained during post-basic nurse training, to a clinical care environment.(10) Communication which impacts on health worker decision making may furthermore take place in 'back stage' ad-hoc opportunistic exchanges, for example in hospital corridors,(8) which can present challenges for capture in research. Despite these challenges for researchers, understanding the ways in which health workers

For peer review only

communicate and work with one another is vital. Understanding 'software' is particularly important in the neonatal unit, where patients often have multiple complex problems that require care by a multi-professional team who are often working across different units (e.g. maternity, neonatal unit, paediatrics).

Since the care of neonates is highly dependent on teams of staff working together, relational ties and social networks are likely to be central to the adoption of new or better care practices, such as those promoted in existing improvement interventions in Kenyan neonatal units. Understanding how and why communication occurs between health workers will help to unpack the many complex influences on staff behaviours and patient care. Furthermore, detailed understanding of causation and explanatory mechanisms will help to identify areas amenable to intervention that are likely to result in desirable change, perhaps overlooked by more traditional research approaches. Through constructing explanatory programme theory, practical opportunities for improving software in neonatal care will be identified.(15)

In preparation for the Pathways Study, a comprehensive realist synthesis of the literature was undertaken, with included data mostly from high-income settings.(16) Findings from this synthesis were used to help guide the focus of scientific enquiry in the Pathways study, and informed the current protocol and study tools. The realist synthesis sought to answer: how, why, for whom, to what extent and in what contexts, do the social ties of hospital staff influence quality of care. The resultant programme theory comprised of 35 context-mechanism-outcome configurations, organised under four emergent thematic domains: Social group, Hierarchy, Bridging distance and Discourse. The Pathways Study will further develop and refine this provisional programme theory for the specific setting of neonatal units in Kenya, using an open and explorative approach.

The findings of the Pathways Study will make an important contribution to the ongoing work of KEMRI-Wellcome Trust Research Programme researchers in Kenya, particularly work focused on improvement, adoption of technologies and staff communication within the neonatal unit, and by exploring neonatal hospital care beyond the neonatal unit. The new

For peer review only - http://bmjopen.bmj.com/site/about/guidelines.xhtml

understanding developed during the Pathways Study will be used for better designing and targeting interventions for quality improvement. Findings will also contribute to existing international literature on social networks of the healthcare workforce, the vast majority of which is derived from high income settings.

Aims and objectives

The aim of the Pathways Study is to explore how, why, for whom and in what circumstances, features of health systems 'software' (e.g. values, norms, relationships) between health workers of all cadres caring for neonates in Kenyan hospitals, influence quality of care being targeted by improvement efforts. The specific objectives are:

I. To describe how health workers of all cadres work together to deliver care to newborn babies in Kenyan hospitals, and the kinds of networks, relationships, social ties and personal/team-related socio-cognitive skills that exist within and between the different groups of health workers caring for newborn babies.

II. To examine how these relationships, networks, social ties and related socio-cognitive skills influence the use of nurses' clinical competencies in newborn care and how these socio-cognitive skills might be utilised to design recommendations to improve neonatal care in Kenyan hospitals.

For peer review only

Methods and analysis

The Pathways study is a realist evaluation,(15,17) which will employ a mixed methods approach to collect relevant data, drawing on diverse methods. Emergent semi-regular patterns will ultimately be identified from these data, to develop iterative explanatory theory for practical use. The initial phase of data collection will be from two hospital case study sites, and from a 'nursing group' (see below). A stakeholder co-design workshop will be convened following the initial analysis, to further refine theory, and to develop recommendations for practice (Figure 1).

Figure 1: Summary of mixed methods used in the Pathways Study

Hospital Case Study Sites

Data will be collected from two large urban public hospitals in Kenya. The study sites have been chosen in part due to ease of access, owing to the existing COVID-19 restrictions to movements. They are also facilities of two contrasts. The Pathways Study will collect data from eligible healthcare workers involved in the provision of care to neonates in the two hospital sites.

Hospital 1 is a large teaching and referral hospital, with a large neonatal unit with a Newborn Intensive Care Unit, and is an implementing site for ongoing initiatives to improve care of neonatal service through the 'NEST360 (2022 online) Delivering and Sustaining Newborn Technologies',(5) that seeks to integrate technological solutions within clinical care. The hospital is a training centre for Basic Diploma Nursing and Clinical Medicine students, Bachelor of Science in Nursing and Bachelor of Medicine and Surgery undergraduate

For peer review only

students, Post-Graduate Diploma, Higher Diploma and Master's level training for Paediatric Nursing, Neonatal Nursing, Paediatrics and Child Health and a Fellowship in Neonatology.

Hospital 2 is a large county hospital, with a smaller neonatal unit. Unlike Hospital 1, Hospital 2 is a member of the 'Clinical Information Network',(18) which seeks to improve clinical care of children by targeting better use of routine clinical data to inform policy and practice through providing audit and feedback mechanisms and supporting the work of clinical champions in participating network hospitals. Unlike Hospital 1, Hospital 2 is not an implementing site for NEST360(5)

Nursing group

In addition to the hospital case studies, qualitative data relevant to exploring socio-cognitive skills and the implementation of learned knowledge and skills in the workplace, will be collected from a purposively sampled nursing graduate group and nurse educators from affiliated nurse training institutions.

Stakeholder group

A group of relevant stakeholders will be invited to participate in a co-design event, following initial data collection and analysis. Co-design outputs will be refined theory and associated recommendations for practice.

For peer review only
For peer review only - http://bmjopen.bmj.com/site/about/guidelines.xhtml

Inclusion and exclusion criteria for participation are shown in table 1 below.

Table 1: Study inclusion and exclusion criteria

Methods of data collection	Inclusion criteria	Exclusion criteria
• Non-participant observation<br>• Social network questionnaire<br>• In-depth interviews	• Must work in the delivery room, post-natal ward, neonatal unit, or paediatric ward, or support the function of these units as part of their work within the wider hospital.<br>• Relevant staff of all cadres and grades will be included, for example nurse, doctor, midwife, nutritionist, clinical officer, patient care attendant, housekeeping, physiotherapist, orthopaedic technician, mortician, kitchen staff, laboratory staff, pharmacy staff, hospital administration.<br>• Nursing and midwifery students and final year medical and clinical officer students will also be included, to reflect their role in service provision.<br>• Must be aged 18 years or over<br>• Must be able to provide informed consent<br>• Must be available to participate in the study, without their participation adversely affecting to the functioning of the neonatal/maternity unit (e.g. before/after shift or during break)	• Does not work in, or provide any support to neonatal care delivery in the hospital<br>• Aged under 18 years<br>• Unable to give informed consent<br>• Unable to participate in study, without participation adversely affecting the delivery of neonatal care

For peer review only

• Key Informant Interviews	Senior Nurse/Manager in the hospital:<br>• Must be above 18 years<br>• Must be duly registered and licensed to practice at the level of Diploma in Nursing and above<br>• Must be an employee of the hospital under study<br>• Must have formal leadership roles (unit manager, team leader, shift leader etc) in the neonatal or paediatric ward<br>• Must be able to provide informed consent<br>• Must be available to participate in the study, without their participation adversely affecting to the functioning of the neonatal unit	• Junior nurses without leadership or management roles<br>• Nurses working on locum or employees of other hospitals/agencies<br>• Aged less than 18 years<br>• Not willing or able to provide informed consent<br>• Unable to participate in the study due to work pressures.
	Nurse Educator in the attached training facility:<br>• Must be above 18 years<br>• Must be duly registered and licensed to practice at the level of Post-basic Diploma in Nursing and above<br>• Must be an employee of the training institution<br>• Must have formal nursing programme administration/management role (head of academic programme, head of nursing thematic programme, But not the college principal or dean or director)<br>• Must be able to provide informed consent<br>• Must be available to participate in the study.	• Junior teaching faculty without programme leadership or management roles<br>• Educators working on part-time or are employees of other hospitals/agencies<br>• Aged less than 18 years<br>• Not willing or able to provide informed consent<br>• Unable to participate in the study due to work pressures.

For peer review only

For peer review only - http://bmjopen.bmj.com/site/about/guidelines.xhtml

• Focus group discussions	Basic nursing graduates:<br>• Must have a diploma or degree in nursing and/or midwifery<br>• Must be registered and licensed by Nursing Council of Kenya to practice in Kenya<br>• Must be above 18 years<br>• Must be able to provide informed consent<br>• Are working in hospitals in Kenya on full-time, part-time or locum basis<br>• Must be available to participate in the study	• Nurses with certificate or post-diploma/post-graduate qualifications in specialty nursing<br>• Pre-service nursing students<br>• Not willing or able to provide informed consent<br>• Unable to participate in the study due to work pressures.
	Nursing graduates: Neonatal and Paediatric Nurses or Maternal-neonatal nurses<br>• Must have a post-basic diploma/post-graduate diploma or MSc in nursing (neonatal or paediatric nursing or maternal-neonatal nursing)<br>• Must be registered (or due for registration) and licensed (or due for licensure) by Nursing Council of Kenya to practice Neonatal or Paediatric Nursing in Kenya<br>• Must be above 18 years<br>• Must be able to provide informed consent<br>• Are working in hospitals in Kenya on full-time, part-time or locum basis<br>• Must be available to participate in the study	• Nurses without specialist training in neonatal, paediatric or maternal-neonatal nursing<br>• In-service nursing students not yet due for registration or licensure as neonatal, paediatric or maternal-neonatal nurses<br>• Not willing or able to provide informed consent<br>• Unable to participate in the study due to work pressures.

For peer review only - http://bmjopen.bmj.com/site/about/guidelines.xhtml

	Nurse educators:<br>• Must have at a minimum, a post-graduate diploma in nursing and/or midwifery and/or nursing/medical education.<br>• Must be registered and licensed by Nursing Council of Kenya to practice in Kenya<br>• Are working at various levels (full-time, part-time or locum basis) at the schools of nursing for Kenya Medical Training College, Kenyatta National Hospital and University of Nairobi.<br>• Must be above 18 years<br>• Must be able to provide informed consent<br>• Must be available to participate in the study	• Educators with certificate or diploma qualifications<br>• Educators working in other colleges/institutes other than those mentioned.<br>• Senior managers without teaching roles<br>• Not willing or able to provide informed consent<br>• Unable to participate in the study due to work pressures.
• Stakeholder workshop	• Must have relevant experience in healthcare (particularly paediatric/neonatal care) delivery, research or healthcare policy/education/decision making, e.g. healthcare worker, health systems researcher, policy maker, health service leader, educator, professional representative, or employee of national or county ministry of health or international health agency (as assessed by KWTRP research colleagues)<br>• Must be familiar with key aspects of intervention design or implementation (as assessed by colleagues in extended research team in Kenya)	• Insufficient relevant experience or familiarity with key aspects of healthcare service delivery, research or policy/decision making (as assessed by KWTRP research colleagues)

For peer review only - http://bmjopen.bmj.com/site/about/guidelines.xhtml

Data collection procedures

We will conduct training for all researchers (including research assistants and study clinicians in the various study sites), including the requirements of their roles and how to collect the data using the study data collection tools. Development of the data collection tools (such as observation checklists, interview guides and social network questionnaire) was informed by our previous realist synthesis of the literature.(16) These data collection tools will be piloted amongst the study researchers and colleagues from KEMRI-Wellcome whose work includes some engagements with CIN hospitals, and adapted prior to the start of data collection. To give feedback on the ongoing research process itself, including the piloting and adapting data collection tools, we will also convene a steering group of invited experts.

During data collection and analysis procedures, researchers will adopt a reflexive approach, i.e. all members of the research team will be encouraged to keep aware of their own positionality and biases. Sampling will be purposive, seeking relevant data. The research team will intentionally seek, share and discuss potential biases throughout the course of the Pathways Study, and will mitigate as much as possible through openness and transparent and well documented processes. Training will help to improve individual self-awareness of researchers. The Principal Investigator and Assistant Research Officer will meet regularly to compare and discuss emerging findings.

Data collection methods are listed below, with additional details provided in Table 2.

For peer review only

Table 2: Data collection procedures

Method	When	Average time taken	Justification
1. Non-participant observation	Observations of 6 shifts per hospital site, as discussed with unit manager, to aim for maximum variation. A sample of hospital meetings will also be observed.	6-8 hours per shift on average, plus 6-8 hours of meetings (total 42-56 hours of non-participant observation per site)	Observational data will be used to explore social ties and networks in the hospitals, and influence on provision of neonatal care<br>**Objective 1**
2. In-depth semi-structured interviews	One interview per participant (up to total 35 in each of 2 hospital sites)	45-60 minutes	In-depth interviews will explore the personal experience and reflections of staff as to how social ties are formed, with whom, and their role and influence in neonatal care provision<br>**Objective 1**
3. Social network questionnaire	One questionnaire per participant (total 40-120+ participants per hospital site, depending on size of networks)	30-45 minutes	Social network data will help to explore the structure of staff communication networks, and network characteristics<br>**Objective 1**
4. Key Informant Interview	One person per each hospital and one person per each attached training facility (Total of 5-7 persons)	30-45 minutes	Key informant interviews will provide additional contextual information<br>**Objective 1**
5. Focus group discussions	Three focus groups (each group of 5-9 members):<br>1) Basic nursing graduates (diploma and degree)<br>2) Specialist neonatal and paediatric nursing graduates<br>3) Nurse educators for both basic and specialised post-basic neonatal or paediatric nursing programmes.	Each focus group lasting 60-90 minutes	Focus group discussions will explore socio-cognitive aspects of utilising knowledge and skills in the workplace<br>**Objective 1**

For peer review only - http://bmjopen.bmj.com/site/about/guidelines.xhtml

6. Stakeholder Workshop	One session following the data analysis from methods 1-3.<br>Total of 8-12 participants	One day (either in-person or online) lasting 5-7 hours	The stakeholder workshop will provide data and use elements of co-design, to develop recommendations for how understanding of social ties could enhance efforts to improve quality of care for newborn babies<br>**Objective 2**

For peer review only

Non-participant observation

In the two hospital case study sites, we will observe health workers in the units/departments they are working. Sampling of shifts (6 per hospital) will be discussed with the unit manager and shifts will be purposively chosen to select a range of different shifts, and to capture variation. A sample of relevant hospital meetings will also be observed at each hospital site. Consent will be sought from staff at the start of the shift, and observations by the researcher will continue throughout the entire shift to try to mitigate any possible changes in staff behaviour due to being observed. Six shifts will be observed per hospital, to also try to mitigate any potential changes in staff behaviour due to being observed. Though not the focus of observations, caregivers will also have the presence of the researcher explained to them and be given the option to opt out of them/their baby being present in observations of staff.

Social network questionnaire

We will invite all health workers to participate (a complete network) who are involved in delivering neonatal care from relevant departments (i.e. neonatal unit, paediatric and maternity wards, and the allied departments: laboratory, pharmacy, radiology, nutrition and patient support services). If the number of staff involved in delivering neonatal care exceeds 120, those staff who are most directly involved in the delivery of neonatal care by their place of work being on the neonatal unit, paediatric or maternity wards, will be invited to participate in the social network questionnaire in the first instance.

A complete staffing list will be compiled by speaking to the management of the hospital/units and relevant student representatives (list will include names of staff of all cadres, students, and all shifts). The researcher will assign a random ID code to each member of staff on the list, using a random sequence generator. Participants will be asked to identify colleagues they would speak to from a staffing list, and the researcher will use a separate research list to locate a corresponding ID code for each member of staff/student named during the social network questionnaire. Demographic data will be collected to identify and better understand homophily (those with similar demographics forming preferential ties with one another) within the network,

For peer review only - http://bmjopen.bmj.com/site/about/guidelines.xhtml

and where it occurs.(9) This will help to understand for example, whether cadre/gender/academic training is associated with connections in the workplace. If conducted online, participants will be asked to name colleagues without using the roster prompt, to avoid electronic sharing of staff lists.

In-depth interviews

We will conduct purposive sampling to obtain data from approximately 35 study participants from each of the participating hospitals (total of approximately 70). We will seek variation of interview participants to identify and explore diversity of views and experience, for example variation in cadres, units, and demographics.

Key Informant Interviews

One representative from management will be invited to participate in a key informant interview, from each of the two hospitals and from associated training institutions.

Focus group discussions

Multi-stage sampling (convenience and snowballing then stratified sampling) will be used for focus group discussions of basic diploma and/or degree nursing graduates, specialist paediatric/neonatal nursing graduates and nursing educators. Participants from training institutions (nursing educators) will be recruited purposively. For nurse graduates, informal/formal professional nursing graduate social media group fora, such as WhatsApp and Telegram, will be used to invite individuals to participate in the study, followed by snowballing to reach those who are not on the social media platforms. This will be followed by stratification (1:1:1) to represent the various professional cadres, training programmes and facilities where they practice.

Stakeholder Workshop

For peer review only - http://bmjopen.bmj.com/site/about/guidelines.xhtml

We will conduct purposive sampling to obtain data from at least one representative amongst the various stakeholders, to achieve a sample size of 10-15.

Analyses

The research objectives of the Pathways Study which aim to build theory, and the mixed methods approach to data collection, make realist evaluation an appropriate scientific approach for analysis and interpretation.

A realist programme theory will be developed, and recommendations for practice will be made from the developed theory. Overaching realist programme theory will be developed through examination of data, initially by seeking out semi-regular patterns of outcomes that occur within the programme theory and then developing causal explanations for these outcomes in the form of context-mechanism-outcome configurations (CMOCs).(19) The initial programme theory (and its consitituent CMOCs) for the Pathways Study will be based on our existing realist review.(16) The data collected will be analysed using the same realist logic of analysis to further develop the programme theory developed from this realist review. Hence, the programme theory from the realist review will initially provide causal explanations that can be 'tested' (confirmed, refuted or refined) against the primary data collected for the realist evalaution.(15) Where indicated by our interpretations of the data, additional CMOCs will be added to the initial programme theory – thus further developing the understanding captured in the programme theory.

Prior to the application of the realist logic of analysis we will do some additional data analysis, for example thematically analysing the qualitative data and using social network approaches to analyse the quantitative data:

- Qualitative data from the study collected during non-participant observations, in-depth interviews and focus groups, and the stakeholder co-design event will initially be analysed thematically using NVivo software. This will lead to inductively

For peer review only - http://bmjopen.bmj.com/site/about/guidelines.xhtml

developing a theoretical framework. Qualitative data on socio-cognitive aspects of the study (key informant interviews and in-depth interviews) will be iteratively analysed using a grounded theory approach following an Open-coding →Axial-coding →Selective-coding approach to develop an explanatory theoretical framework on the role of socio-cognitive skills in the utilization of clinical competencies by nurses.

- Quantitative data collected from a Social Network Questionnaire will be analysed with standard SNA metrics (e.g. density, centrality, etc.) using Gephi free software and/or R to develop sociograms and a set of network measures.

Following the initial phase of realist anlysis and theory building, a group of stakeholders will be invited to review and further refine the theory, based on their expert knowledge and experiences. Through engagement with these stakeholders, explanatory theory will be further refined, and used to develop a set of recommendations for practice.

Patient and Public Involvement

The Pathways Study research team includes a representative from each of the two hospital study sites and training institutions as study collaborators. The role of study site collaborators is to provide useful linkages for facility engagements before, during and after the study as well as facilitate the smooth conduct of the research by contributing to data collection through arranging for meetings with study participants. During the study, an advisory steering group will be formed to review study findings and provide useful comments and expert advisory on study data analysis and interpretation of the study findings.

For peer review only - http://bmjopen.bmj.com/site/about/guidelines.xhtml

Ethics and dissemination

Ethical approval for the Pathways Study has been received from Kenya Medical Research Institute (KEMRI/SERU/CGMR-C/241/4374) and Oxford Tropical Research Ethics Committee (OxTREC 519-22).

Insights from the Pathways Study will add new understanding and a different theoretical lens to ongoing work by KEMRI-Wellcome Trust Research Programme in Kenya, particularly work focused on health workforce communication in neonatal care. Findings will also provide a useful addition to the existing body of literature on social networks of the hospital workforce, which is almost entirely derived from high income settings. The explanatory power of realist programme theory in specifically developing new understanding around the chains of causation associated with social ties of hospital staff, will be useful for designing and targeting interventions to enhance how such ties might positively contribute to change efforts in the neonatal unit. In addition, social network analysis (SNA) is a relatively new methodology in healthcare research, and so the practical learning gained during the research process will be shared with KEMRI-Wellcome Trust Research Programme colleagues and others and will be useful for those intending to use SNA in future research.

Findings will further open-up the window of socio-cognitive skills to policymakers and other stakeholders in Kenya, their role in service delivery quality and by way of interventions influence health workforce socio-cognitive skills strengthening agendas. Findings relating to the role of non-technical socio-cognitive skills in the use of learned clinical competencies in neonatal care, will be used to develop recommendations for nurse educational interventions. Recommendations are likely to include how and when these skills can be incorporated into basic and specialised nursing programmes and in-service continuous professional development training activities. We anticipate that these findings might also be applicable to wider healthcare training programmes.

For peer review only

For peer review only - http://bmjopen.bmj.com/site/about/guidelines.xhtml

Findings will be shared with the two study hospitals, relevant educational institutions, and KEMRI-WELLCOME Trust Research Programme and the University of Oxford. Study findings will also be disseminated in seminars, local and international conferences, and as academic theses and research articles published in open access scientific journals.

For peer review only

For peer review only - http://bmjopen.bmj.com/site/about/guidelines.xhtml

Authors' contributions

This protocol was co-developed by Claire Blacklock (CB) and Conrad Wanyama (CW) as joint authors/co-principal investigators. Mike English (ME), Peris Musitia (PM), Mwanamvua Boga (MB), Geoff Wong (GW), Lisa Hinton (LH) contributed to the development of the protocol, and Juliet Jepkosgei (JJ) contributed to the refining of the protocol and study tools for implementation. We received protocol review feedback from Michuki Maina (MM), Betty Kalama (BK) and Naomi Muinga (NM), who were nominated as KEMRI- Coast Centre Committee (CCC) and Alex Hinga and Nancy Kagwanja as KEMRI Centre Scientific Committee reviewers. Both the CCC and CSC review feedbacks were incorporated in the final protocol.

Funding statement

The Pathways Study is supported by the Wellcome Trust (#207522) through an award to ME as a Senior Fellowship.

CB is studying for a DPhil - this work is supported by the Nuffield Department of Medicine, University of Oxford and the Medical Research Council [grant number MR/N013468/1].

The funders had no role in the preparation of this report or the decision to submit for publication.

Open Access Policy

This research is funded in whole or in part by the Wellcome Trust (#207522). For the purpose of Open Access, the author has applied a CC-BY public copyright licence to any author accepted manuscript version arising from this submission

For peer review only - http://bmjopen.bmj.com/site/about/guidelines.xhtml

Competing interests statement

All authors declare no competing or conflict of interests.

For peer review only

For peer review only - http://bmjopen.bmj.com/site/about/guidelines.xhtml

References

1. United Nations Inter-Agency Group for Child Mortality Estimation (UN IGME). Levels & trends in child mortality. Report 2019. New York: UNICEF, 2019. Available at https://childmortality.org/wp- content/uploads/2019/09/UN-IGME-Child-Mortality-Report-2019.pdf

2. Hug L, Alexander M, You D, Alkema L. National, regional, and global levels and trends in neonatal mortality between 1990 and 2017, with scenario-based projections to 2030: a systematic analysis. Lancet Glob Heal. 2019;7(6):e710–20.

3. Keene CM, Aluvaala J, Murphy GAV, Abuya N, Gathara D, English M. Developing recommendations for neonatal inpatient care service categories: Reflections from the research, policy and practice interface in Kenya. BMJ Glob Heal. 2019;4(2).

4. Maina M, Aluvaala J, Mwaniki P, Tosas-Auguet O, Mutinda C, Maina B, et al. Using a common data platform to facilitate audit and feedback on the quality of hospital care provided to sick newborns in Kenya. BMJ Glob Heal. 2018;3(5):e001027.

5. NEST360 (2022 online). Delivering and Sustaining Newborn Technologies. Available at https://nest360.org/technology/

6. Sheikh K, Gilson L, Agyepong IA, Hanson K, Ssengooba F, Bennett S. Building the field of health policy and systems research: Framing the questions. PLoS Med. 2011;8(8):1–6.

7. Scott J, Revera Morales D, Mcritchie A, Riviello R, Smink D, Yule S. Non-technical skills and health care provision in low- and middle-income countries: A systematic review. Med Educ. 2016;50(4):441–55.

8. Lewin S, Reeves S. Enacting "team" and "teamwork": Using Goffman's theory of impression management to illuminate interprofessional practice on hospital wards. Soc Sci Med. 2011;72(10):1595–602.

For peer review only - http://bmjopen.bmj.com/site/about/guidelines.xhtml

9. Scott, J. Social network analysis. 2nd ed. Los Angeles: SAGE Publications Ltd, 2000.

10. Rogers EM. Diffusion of Innovations. New York: Free Press; 2003.

11. De Herdt T, de Sardan J. Real governance and practical norms in sub-Saharan Africa. Routledge; 2015.

12. Liberati EG, Tarrant C, Willars J, Draycott T, Winter C, Chew S, et al. How to be a very safe maternity unit: An ethnographic study. Soc Sci Med.. 2019;223(January):64–72.

13. Liberati EG, Tarrant C, Willars J, Draycott T, Winter C, Kuberska K, et al. Seven features of safety in maternity units: a framework based on multisite ethnography and stakeholder consultation. BMJ Qual Saf. 2020;bmjqs-2020-010988.

14. Prineas S, Mosier K, Mirko C, Guicciardi S. Non-technical Skills in Healthcare BT - Textbook of Patient Safety and Clinical Risk Management. In: Donaldson L, Ricciardi W, Sheridan S, Tartaglia R, editors. Cham: Springer International Publishing; 2021. p. 413–34.

15. Pawson, R, Tilley, N. Realistic evaluation. London: SAGE Publications Ltd, 1997

16. Blacklock C, Darwin A, English M, McKnight J, Hinton L, Harris E, Wong G. The social networks of hospital staff: a realist synthesis. J Health Serv Res Policy. 2022;0(0):1–11.

17. Wong G, Westhorp G, Manzano A, Greenhalgh J, Jagosh J, Greenhalgh T. RAMESES II reporting standards for realist evaluations. BMC Med. 2016;14(1):1–18.

18. Tuti T, Bitok M, Malla L, Paton C, Muinga N, Gathara D, et al. Improving documentation of clinical care within a clinical information network: An essential initial step in efforts to understand and improve care in Kenyan hospitals. BMJ Glob Heal. 2016;1(1):1–8.

For peer review only - http://bmjopen.bmj.com/site/about/guidelines.xhtml

19. Wong G, Westhorp G, Pawson R, Greenhalgh T. Realist Synthesis. RAMESES Training Materials. RAMESES Proj. 2013;(July 2013):55.

For peer review only

Figure legend

Figure 1: A summary of mixed methods used in the Pathways Study

The figure describes the methodological approach for data collection in phase 1 (in the first three horizontal boxes), which involve data collection from the two case study hospitals and additional data from a nursing interest group, that will comprise nurses providing neonatal nursing care in the two case study hospitals and possibly other hospitals within Kenya. During this phase 1 work, we will also form a technical group for defining a realist evaluation programme theory. The last box, below the 3 boxes, describes a phase 2 data collection, where study findings from the phase 1 study participants as well as the initially developed programme theory, will be discussed over a one-day workshop by a group of prior nominated stakeholders. These stakeholders will be drawn from memberships of neonatal nurses, neonatal and paediatric consultants, neonatal and paediatric professional associations, Kenya's Ministry of Health, division of newborn care, Kenya's medical and nursing practice regulatory agencies and senior health systems researchers from KEMRI-Wellcome Trust Research Programme. It is the recommendations from these stakeholder group that will inform the design of interventions targeting behaviour change among health workers providing care to neonates in Kenya.

For peer review only

Figure 1: Summary of mixed methods used in the Pathways Study

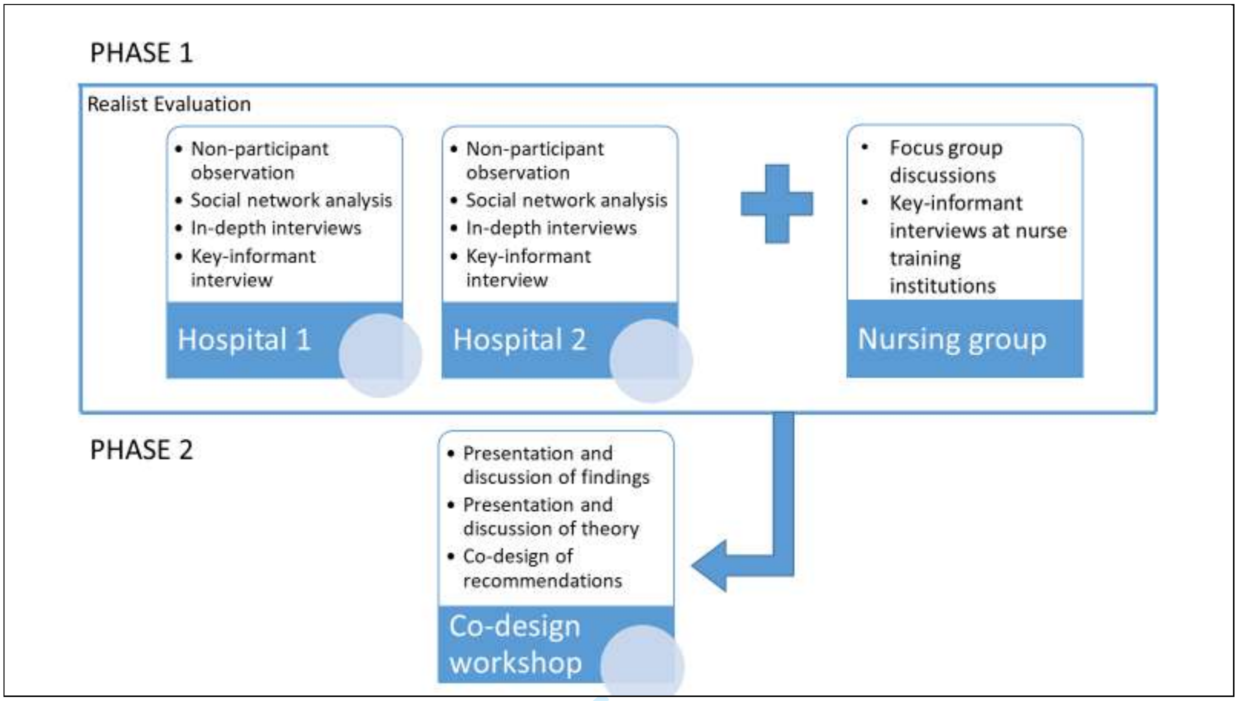

For peer review only

BMJ Open

Protocol for the Pathways Study: a realist evaluation of staff social ties and communication in the delivery of neonatal care in Kenya

Journal:	*BMJ Open*
Manuscript ID	bmjopen-2022-066150.R1
Article Type:	Protocol
Date Submitted by the Author:	24-Jan-2023
Complete List of Authors:	Wanyama, Conrad; KEMRI-Wellcome Trust Research Programme<br>Blacklock, Claire; University of Oxford, Nuffield Department of Medicine<br>Jepkosgei, Juliet; KEMRI-Wellcome Trust Research Programme<br>English, Mike; Univerity of Oxford Nuffield Department of Medicine, Nuffield Department of Medicine and Department of Paediatrics; KEMRI-Wellcome Trust Research Programme Nairobi,<br>Hinton, Lisa; University of Cambridge, The Healthcare Improvement Studies Institute<br>McKnight, Jacob ; Univerity of Oxford Nuffield Department of Medicine, Tropical Medicine; University of Oxford Nuffield Department of Clinical Medicine<br>Molyneux, Sassy ; University of Oxford, Nuffield Department of Medicine; KEMRI-Wellcome Trust Research Programme, Centre for Geographic Medicine Research-Coast<br>Boga, Mwanamvua ; KEMRI-Wellcome Trust Research Programme, Centre for Geographic Medicine Research-Coast<br>Musitia, Peris; KEMRI-Wellcome Trust Research Programme Nairobi, Health Services Unit; KEMRI-Wellcome Trust Research Programme Nairobi, Health Services Unit<br>Wong, Geoff; Oxford University, Nuffield Department of Primary Care Health Sciences
**Primary Subject Heading**:	Communication
Secondary Subject Heading:	Communication, Medical education and training, Research methods
Keywords:	Health policy < HEALTH SERVICES ADMINISTRATION & MANAGEMENT, Quality in health care < HEALTH SERVICES ADMINISTRATION & MANAGEMENT, NEONATOLOGY

SCHOLARONE™
Manuscripts

Protocol for the Pathways Study: a realist evaluation of staff social ties and communication in the delivery of neonatal care in Kenya

Wanyama C*[1], Blacklock C*[2], Jepkosgei J[1], English M[1,2], Hinton L[3], McKnight J[2], Molyneux S[1,2], Boga M[1], Musitia P[1], Wong G[4].

*Joint first authors

[1]Health Services Unit, KEMRI-Wellcome Trust Research Programme, Nairobi, Kenya

[2]Nuffield Department of Medicine, University of Oxford, UK

[3]The Healthcare Improvement Studies Institute, University of Cambridge, UK

[4]Nuffield Department of Primary Care Health Sciences, University of Oxford, UK

Corresponding author:

Conrad Wanyama[1],

KEMRI-Wellcome Trust Research Programme

P.O Box 43640-00100

Nairobi, Kenya

cwanyama@kemri-wellcome.org

For peer review only - http://bmjopen.bmj.com/site/about/guidelines.xhtml

Abstract

Introduction: The informal social ties that health workers form with their colleagues influence knowledge, skills and individual and group behaviours and norms in the workplace. However, improved understanding of these 'software' aspects of the workforce (e.g. relationships, norms, power) have been neglected in health systems research. In Kenya, neonatal mortality has lagged despite reductions in other age groups under 5yrs. A rich understanding of workforce social ties is likely to be valuable to inform behavioural change initiatives seeking to improve quality of neonatal healthcare.

This study aims to better understand the relational components among health workers in Kenyan neonatal care areas, and how such understanding might inform the design and implementation of quality improvement interventions targeting health workers' behaviours.

Methods and analysis: We will collect data in two phases. In Phase One we will conduct non-participant observation of hospital staff during patient care and hospital meetings, a social network questionnaire with staff, in-depth interviews, key informant interviews, and focus group discussions at 2 large public hospitals in Kenya. Data will be collected purposively and analysed using realist evaluation, interim analyses including thematic analysis of qualitative data and quantitative analysis of social network metrics. In Phase Two, a stakeholder workshop will be held to discuss and refine phase one findings.

Study findings will help refine an evolving programme theory with recommendations used to develop theory-informed interventions targeted at enhancing quality improvement efforts in Kenyan hospitals.

Ethics and dissemination: The study has been approved by Kenya Medical Research Institute (KEMRI/SERU/CGMR-C/241/4374) and Oxford Tropical Research Ethics Committee (OxTREC 519-22). Research findings will be shared with the sites, and, disseminated in seminars, conferences, and, published in open-access scientific journals.

For peer review only - http://bmjopen.bmj.com/site/about/guidelines.xhtml

Strengths and limitations of this study

Strengths

- Realist evaluation is an appropriate methodology to develop middle-range programme theory, which will be useful for informing the practical design and implementation of quality improvement interventions in neonatal units.
- The Pathways Study is the first to use social network analysis to explore the influence of staff social ties on the delivery and quality of neonatal care in Kenya.
- Middle-range programme theory will be developed using a robust and transparent process, using an initial programme theory, involvement of stakeholders, substantive sociological theory, and continual collaborative and reflexive working within a diverse research team.

Limitations

- The Pathways Study uses a mixed methods approach to collect diverse data, but it is possible that some relevant data may still not be captured, particularly as some aspects of health systems 'software' are notoriously difficult to elicit.

For peer review only

For peer review only - http://bmjopen.bmj.com/site/about/guidelines.xhtml

Introduction

Despite significant reductions in mortality rates across other age groups among children under-5 in recent years, the neonatal mortality rate in Kenya has remained relatively unchanged.(1,2) In response to this disparity, efforts are ongoing to improve the quality of neonatal care, the majority of which is delivered from public health facilities and mostly by nurses.(3) Such improvement efforts have included the recent introduction of new technologies in selected neonatal units, establishing routine neonatal data collection and feedback systems, and ongoing investment in the neonatal workforce through training and workplace support.(4,5)

However, a more detailed understanding of tacit human factors, or health systems 'software',(6) that also influence the quality of neonatal care delivered in these hospitals is now needed, to inform better focused design and implementation of improvement efforts (e.g. the relationships between staff, values, norms and the social and cognitive skills that complement clinical technical skills).(7) 'Software' in health systems (in contrast to 'hardware', such as drugs, equipment, number of staff etc.) has been defined as "the ideas and interests, values and norms, and affinities and power that guide actions and underpin the relationships among system actors and elements".(6) Indeed, such 'software' factors can profoundly influence acquisition of new knowledge and behaviours and norms at individual and group level,(8–11) and thus are determinants of quality care.(12,13) Moreover, individual health worker performance is shaped not only by technical clinical competencies and experiences, but also by social and cognitive skills (cognitive, social and personal resource skills such as communication, team work, leadership, situational awareness, assertiveness and decision making, coping with stress and managing fatigue etc).(14) Relational ties among the workforce and other 'software' aspects matter greatly in the delivery of quality neonatal care, for example care pathways, clinical guidelines, knowledge, and other competencies, including those that are transferred from an educational setting such as those gained during post-basic nurse training, to a clinical care environment.(10) The impact of relational ties on clinical decision

For peer review only - http://bmjopen.bmj.com/site/about/guidelines.xhtml

making was demonstrated in the landmark study by Coleman et al. (15) showing profound peer influence on prescribing of a new drug by physicians in the United States of America. More recent studies have likewise demonstrated peer group influence on both clinical practices and association with differences in patient outcome. (16-18) Communication which impacts on health worker decision making may furthermore take place in 'back stage' ad-hoc opportunistic exchanges, for example in hospital corridors,(8) which can present challenges for capture in research. Despite these challenges for researchers, understanding the ways in which health workers communicate and work with one another is vital. Better understanding of healthcare 'software' is particularly important in the neonatal unit, where patients often have multiple complex problems that require care by a multi-professional team who are often working across different units (e.g. maternity, neonatal unit, paediatrics).

Since the care of neonates is highly dependent on teams of staff working together, relational ties and social networks are likely to be central to the adoption of new or better care practices, such as those promoted in existing improvement interventions in Kenyan neonatal units. Understanding how and why communication occurs between health workers will help to unpack the many complex influences on staff behaviours and patient care. Furthermore, detailed understanding of causation with a focus on explanatory 'mechanisms' (*Box 1: Definitions of Key Terms*) will help to identify aspects of 'context' (Box 1) amenable to intervention that are likely to result in desirable change in 'outcome' (Box 1), perhaps overlooked by more traditional research approaches. Through developing explanatory middle-range programme theory (Box 1), practical opportunities for improving software in neonatal care will be identified.(19,20)

For peer review only

Box 1: Definitions of key terms (19,21)

**Context:**
A specific aspect of the setting/environment of study, which when present triggers the activation of a mechanism(s).
**Mechanism:**
A latent (often invisible) property/entity, sensitive to variations in context, which when activated causes an outcome to occur.
**Outcome:**
A desirable (or undesirable) event/occurrence, which is of interest by its presence or absence (or strength/degree of).
**Context-mechanism-outcome configuration:**
A unit/statement/diagram which links and orders a context, mechanism and outcome, to provide causal explanation. It is often abbreviated to CMO or CMOC.
**Middle-range programme theory:**
The product of realist enquiry – an abstracted theory derived from relevant and robust data, which is both testable, and transferable to other settings by application of abstracted learning.
**Initial programme theory:**
A rough outline of a theory of causation pertaining to the question under study, to guide the process of investigation.
**Demi-regularity:**
An observable pattern in the data, which occurs in certain circumstances.
**Retroduction:**
Intentional consideration, exploration and identification of the hidden elements of causation that lie beneath observable data and logic.

In preparation for the current Pathways Study described in this manuscript, a comprehensive realist synthesis of the literature was undertaken to provide an initial programme theory (22) which was used to help guide the focus of scientific enquiry in the Pathways study, and inform the current protocol and study tools, through generation of an initial theory to be tested and further developed. The preparatory realist synthesis hence sought to answer: how, why, for whom, to what extent and in what contexts, do the social ties of hospital staff influence quality

For peer review only - http://bmjopen.bmj.com/site/about/guidelines.xhtml

of care. Details of how the initial programme theory was developed can be found in the published realist synthesis – in brief, comprehensive literature searching, reviewing, data extraction, analysis and interpretation, was informed by engagement with stakeholders with relevant expertise and experience, from Sierra Leone and Kenya. The resultant theory comprised of 35 context-mechanism-outcome configurations, organised under four emergent thematic domains: Social group, Hierarchy, Bridging distance and Discourse (*see Box 2: Summary of initial programme theory*). The Pathways Study will further develop and refine this initial programme theory for the specific setting of neonatal units in Kenya, using an open and explorative approach, cognisant that the majority of data used to produce the initial programme theory were from high-income settings. (22)

Box 2: Summary of initial programme theory - taken from Blacklock et al. JHSRP 2022 (22)

**Initial Programme Theory, to be tested (confirmed, refuted, refined) in the Pathways Study.**
Hospital staff prefer to communicate with colleagues who are similar to themselves, and with whom they share trust. However, this can create boundaries, silos and redundant information within pockets of the workforce, and different behavioural norms adopted by members of different groups, dominated by influential individuals. Tacit hierarchical rules also determine communication with others in the workplace based on status, forming a landscape of differential access to information and others. Fragmentation between different status groups can occur, and space for status-enhancing behaviours is created. Workplace silos and status boundaries can be bridged in the presence of trust, confidence and mutual understanding, or where need is urgent and immediate. The organisation can actively comply with or challenge social boundaries within the workforce by the formal processes endorsed, which allow day-to-day performances of relative power, based on identity and hierarchical status. Attempts to manage the discourse, and the effectiveness of formal workplace communication processes, act to construct or disassemble social boundaries in hospitals. Connections between staff influence access to information and other forms of capital, behavioural norms and perceived agency. Ties are continuously shaped and as such are amenable to intervention. The capacity of these connections and the overall structure of the workplace social network are vital to delivering quality patient care.

The findings of the Pathways Study will make an important contribution to the ongoing work of KEMRI-Wellcome Trust Research Programme researchers in Kenya, particularly work focused on improvement, adoption of technologies and staff communication within the neonatal unit, and by exploring neonatal hospital care beyond the neonatal unit. The new

For peer review only - http://bmjopen.bmj.com/site/about/guidelines.xhtml

understanding developed during the Pathways Study will be used for better designing and targeting interventions for quality improvement. Findings will also contribute to existing international literature on social networks of the healthcare workforce, the vast majority of which is derived from high income settings.

Aims and objectives

The aim of the Pathways Study is to explore how, why, for whom and in what circumstances, features of health systems ‘software’ (e.g. values, norms, relationships) between health workers of all cadres caring for neonates in Kenyan hospitals, influence quality of care being targeted by improvement efforts. The specific objectives are:

I. To describe how health workers of all cadres work together to deliver care to newborn babies in Kenyan hospitals, and the kinds of networks, relationships, social ties and personal/team-related socio-cognitive skills that exist within and between the different groups of health workers caring for newborn babies.

II. To examine how these relationships, networks, social ties and related socio-cognitive skills influence nurses’ clinical competencies in newborn care and how these socio-cognitive skills might be utilised to design recommendations to improve neonatal care in Kenyan hospitals.

For peer review only - http://bmjopen.bmj.com/site/about/guidelines.xhtml

Methods and analysis

The Pathways study is a realist evaluation,(20,23) which will employ a mixed methods approach to collect relevant data, drawing on diverse methods. (21) Emergent semi-regular patterns (i.e. demi-regularities, Box 1) and causative mechanisms will ultimately be identified from these data using a process of retroduction (Box 1), to develop iterative explanatory theory for practical use. An initial programme theory derived from a realist synthesis of the literature (22) has been used to guide design of data collection tools for the Pathways Study and will also form a starting point for analysis and data-informed theory refinement. The first phase of data collection will be from two hospital case study sites, and from a ‘nursing group’ (see below). A stakeholder co-design workshop will then be convened following the initial analysis, to further refine middle-range programme theory, and to develop recommendations for practice (phase 2, Figure 1).

Figure 1: Summary of mixed methods used in the Pathways Study

For peer review only

Hospital Case Study Sites

Data will be collected from two large urban public hospitals in Kenya. The study sites have been chosen in part due to ease of access, owing to the existing COVID-19 restrictions to movements. They are also facilities of contrasts with regards to setting (see below). The Pathways Study will collect data from eligible healthcare workers involved in the provision of care to neonates in the two hospital sites.

Hospital 1 is a large teaching and referral hospital, with a large neonatal unit with a Newborn Intensive Care Unit, and is an implementing site for ongoing initiatives to improve care of neonatal service through the ‘NEST360 (5), Delivering and Sustaining Newborn Technologies’

For peer review only - http://bmjopen.bmj.com/site/about/guidelines.xhtml

that seeks to integrate technological solutions within clinical care. The hospital is a training centre for Basic Diploma Nursing and Clinical Medicine students, Bachelor of Science in Nursing and Bachelor of Medicine and Surgery undergraduate students, Post-Graduate Diploma, Higher Diploma and Master's level training for Paediatric Nursing, Neonatal Nursing, Paediatrics and Child Health and a Fellowship in Neonatology.

Hospital 2 is a large county hospital, with a smaller neonatal unit. Unlike Hospital 1, Hospital 2 is a member of the 'Clinical Information Network' (CIN),(24) which seeks to improve clinical care of children by targeting better use of routine clinical data to inform policy and practice through providing audit and feedback mechanisms and supporting the work of clinical champions in participating network hospitals. Unlike Hospital 1, Hospital 2 is not an implementing site for NEST360(5)

Nursing group

In addition to the hospital case studies, qualitative data relevant to exploring socio-cognitive skills and the implementation of learned knowledge and skills in the workplace, will be collected from a purposively sampled nursing graduate group and nurse educators from affiliated nurse training institutions.

Stakeholder group

A group of 8-12 relevant stakeholders will be invited to participate in a co-production event, following initial data collection and analysis. Co-produced outputs will be refined theory and associated recommendations for practice. The invited stakeholders will be chosen from amongst the eligible groups shown in Table 1, based on relevant knowledge and experience, and convenience.

Inclusion and exclusion criteria for participation are shown in table 1 below.

For peer review only - http://bmjopen.bmj.com/site/about/guidelines.xhtml

Table 1: Study inclusion and exclusion criteria

Data collection	Inclusion criteria	Exclusion criteria
• Non-participant observation<br>• Social network questionnaire<br>• In-depth interviews	• Must work in the delivery room, post-natal ward, neonatal unit, or paediatric ward, or support the function of these units as part of their work within the wider hospital.<br>• Relevant staff of all cadres and grades will be included: nurse, doctor, midwife, nutritionist, clinical officers, non-clinical and support staff, hospital administration, Nursing, midwifery and final year medical and clinical officer students in the mentioned units<br>• Must be aged 18 years or over, be able to provide informed consent and available to participate in the study	• Does not work in, or provide any support to neonatal care delivery in the hospital<br>• Aged under 18 years, unable to give informed consent or unable to participate in study, without participation adversely affecting care delivery
• Key Informant Interviews	Senior Nurse/Manager in the hospital:<br>• Must be above 18 years and be able to provide informed consent<br>• Must be duly registered and licensed to practice at Diploma in Nursing and above<br>• Must be an employee of the hospital under study<br>• Must have formal leadership roles in the neonatal or paediatric ward<br>• Must be available to participate in the study, without their participation adversely affecting to the functioning of the neonatal unit	• Junior nurses without leadership or management roles<br>• Nurses working on locum or other agencies<br>• Aged below 18 years, not willing or able to provide informed consent or unable to participate in the study due to work pressures.
	Nurse Educator in the attached training facility:<br>• Must be above 18 years, able to provide informed consent and available to participate in the study.<br>• Must be duly registered and licensed at Post-basic Diploma in Nursing and above<br>• Must be an employee of the training institution<br>• Must have formal nursing programme administration/management role (head of academic programme, head of nursing thematic programme, But not the college principal or dean or director)	• Junior teaching faculty without leadership or management roles<br>• Educators working on part-time or are employees of other agencies<br>• Aged below 18 years or not willing or able to give informed consent<br>• Unable to participate in the study due to work pressures.

For peer review only - http://bmjopen.bmj.com/site/about/guidelines.xhtml

• Focus group discussions	Basic nursing graduates:<br>• Must have a diploma or degree in nursing and/or midwifery<br>• Must be registered and licensed by Nursing Council of Kenya to practice in Kenya<br>• Must be above 18 years and be able to provide informed consent<br>• Are working in hospitals in Kenya on full-time, part-time or locum basis<br>• Must be available to participate in the study	• Nurses with certificate or post-graduate specialty nurse training<br>• Not willing or able to provide informed consent<br>• Unable to participate in the study due to work pressures.
	Nursing graduates: Neonatal and Paediatric Nurses or Maternal-neonatal nurses<br>• Must have specialty training in neonatal or paediatric or maternal-neonatal nursing and registered/licensed by Nursing Council of Kenya<br>• Must be above 18 years<br>• Must be able to provide informed consent<br>• Are working in hospitals in Kenya on full-time, part-time or locum basis<br>• Must be available to participate in the study	• Nurses without specialty neonatal, paediatric or maternal-neonatal nursing<br>• Not willing or able to provide informed consent<br>• Unable to participate in the study due to work pressures.
	Nurse educators:<br>• Must have at a minimum, a post-graduate diploma in nursing/midwifery/medical education and registered and licensed by Nursing Council of Kenya<br>• Are working full-time or part-time at the schools of nursing for Kenya Medical Training College, Kenyatta National Hospital and University of Nairobi.<br>• Must be above 18 years, be able to provide informed consent and available to participate in the study	• Nurses with certificate or diploma training<br>• Nurses working in other institutes other than study sites or without teaching roles<br>• Not willing or able to provide informed consent or to participate in the study due to work pressures.
• Stakeholder workshop	• Must have relevant experience in healthcare (paediatric/neonatal care) delivery, research or healthcare policy/education/decision making (health systems researcher, policy maker, health service leader, educator, professional representative, or employee of national or county ministry of health or international health agency as assessed by KWTRP)<br>• Must be familiar with key aspects of intervention design or implementation (as assessed by colleagues in extended research team in Kenya)	• Insufficient relevant experience or familiarity with key aspects of healthcare service delivery, research or policy/decision making (as assessed by KWTRP research colleagues)

For peer review only - http://bmjopen.bmj.com/site/about/guidelines.xhtml

Data collection procedures

We will conduct training for all researchers (including research assistants and study clinicians in the various study sites), including the requirements of their roles and how to collect the data using the study data collection tools (study tools attached as appendix a-f, supplementary material) . Development of the data collection tools (such as observation checklists, interview guides and social network questionnaire) was informed by our previous realist synthesis of the literature.(22) These data collection tools will be piloted amongst the study researchers and colleagues from KEMRI-Wellcome whose work includes some engagements with CIN hospitals, and adapted prior to the start of data collection. To give feedback on the ongoing research process itself, including the piloting and adapting data collection tools, we will also convene a steering group of invited experts.

During data collection and analysis procedures, researchers will adopt a reflexive approach, i.e. all members of the research team will be encouraged to keep aware of their own positionality and biases. Sampling will be purposive, seeking relevant data. The research team will intentionally seek, share and discuss potential biases throughout the course of the Pathways Study, and will mitigate as much as possible through openness and transparent and well documented processes. Training will help to improve individual self-awareness of researchers. The Principal Investigator and Assistant Research Officer will meet regularly to compare and discuss emerging findings.

Data collection methods are listed below, with additional details provided in Table 2.

For peer review only - http://bmjopen.bmj.com/site/about/guidelines.xhtml

Table 2: Data collection procedures

Method	When	Average time taken	Justification
1. Non-participant observation	Observations of 6 shifts per hospital site, as discussed with unit manager, to aim for maximum variation. A sample of hospital meetings will also be observed.	6-8 hours per shift on average, plus 6-8 hours of meetings (total 42-56 hours of non-participant observation per site)	Observational data will be used to explore social ties and networks in the hospitals, and influence on provision of neonatal care<br>**Objective 1**
2. In-depth semi-structured interviews	One interview per participant (up to total 35 in each of 2 hospital sites)	45-60 minutes	In-depth interviews will explore the personal experience and reflections of staff as to how social ties are formed, with whom, and their role and influence in neonatal care provision<br>**Objective 1**
3. Social network questionnaire	One questionnaire per participant (total 40-120+ participants per hospital site, depending on size of networks)	30-45 minutes	Social network data will help to explore the structure of staff communication networks, and network characteristics<br>**Objective 1**
4. Key Informant Interview	One person per each hospital and one person per each attached training facility (Total of 5-7 persons)	30-45 minutes	Key informant interviews will provide additional contextual information<br>**Objective 1**
5. Focus group discussions	Three focus groups (each group of 5-9 members):<br>1) Basic nursing graduates (diploma and degree)<br>2) Specialist neonatal and paediatric nursing graduates<br>3) Nurse educators for both basic and specialised post-basic neonatal or paediatric nursing programmes.	Each focus group lasting 60-90 minutes	Focus group discussions will explore socio-cognitive aspects of utilising knowledge and skills in the workplace<br>**Objective 1**

For peer review only - http://bmjopen.bmj.com/site/about/guidelines.xhtml

6. Stakeholder Workshop	One session following the data analysis from methods 1-3.<br><br>Total of 8-12 participants	One day (either in-person or online) lasting 5-7 hours	The stakeholder workshop will provide data and use elements of co-design, to develop recommendations for how understanding of social ties could enhance efforts to improve quality of care for newborn babies<br><br>**Objective 2**

For peer review only

For peer review only - http://bmjopen.bmj.com/site/about/guidelines.xhtml

Non-participant observation

In the two hospital case study sites, we will observe health workers in the units/departments they are working. Sampling of shifts (6 per hospital) will be discussed with the unit manager and shifts will be purposively chosen to select a range of different shifts, and to capture variation. A sample of relevant hospital meetings will also be observed at each hospital site. Consent will be sought from staff at the start of the shift, and observations by the researcher will continue throughout the entire shift to try to mitigate any possible changes in staff behaviour due to being observed. Six shifts will be observed per hospital, to also try to mitigate any potential changes in staff behaviour due to being observed. Though not the focus of observations, caregivers will also have the presence of the researcher explained to them and be given the option to opt out of them/their baby being present in observations of staff.

Social network questionnaire

We will invite all health workers to participate (a complete network) who are involved in delivering neonatal care from relevant departments (i.e. neonatal unit, paediatric and maternity wards, and the allied departments: laboratory, pharmacy, radiology, nutrition and patient support services). If the number of staff involved in delivering neonatal care exceeds 120, those staff who are most directly involved in the delivery of neonatal care by their place of work being on the neonatal unit, paediatric or maternity wards, will be invited to participate in the social network questionnaire in the first instance.

A complete staffing list will be compiled by speaking to the management of the hospital/units and relevant student representatives (list will include names of staff of all cadres, students, and all shifts). The researcher will assign a random ID code to each member of staff on the list, using a random sequence generator. Participants will be asked to identify colleagues they would speak to from a staffing list, and the researcher will use a separate research list to locate a corresponding ID code for each member of staff/student named during the social network questionnaire. Demographic data will be collected to identify and better understand homophily (those with similar demographics forming preferential ties with one another) within the network,

For peer review only - http://bmjopen.bmj.com/site/about/guidelines.xhtml

and where it occurs.(9) This will help to understand for example, whether cadre/gender/academic training is associated with connections in the workplace. If conducted online, participants will be asked to name colleagues without using the roster prompt, to avoid electronic sharing of staff lists.

In-depth interviews

We will conduct purposive sampling to obtain data from approximately 35 study participants from each of the participating hospitals (total of approximately 70). We will seek variation of interview participants to identify and explore diversity of views and experience, for example variation in cadres, units, and demographics.

Key Informant Interviews

One representative from management will be invited to participate in a key informant interview, from each of the two hospitals and from associated training institutions.

Focus group discussions

Multi-stage sampling (convenience and snowballing then stratified sampling) will be used for focus group discussions of basic diploma and/or degree nursing graduates, specialist paediatric/neonatal nursing graduates and nursing educators. Participants from training institutions (nursing educators) will be recruited purposively. For nurse graduates, informal/formal professional nursing graduate social media group fora, such as WhatsApp and Telegram, will be used to invite individuals to participate in the study, followed by snowballing to reach those who are not on the social media platforms. This will be followed by stratification (1:1:1) to represent the various professional cadres, training programmes and facilities where they practice.

Stakeholder Workshop

For peer review only - http://bmjopen.bmj.com/site/about/guidelines.xhtml

We will conduct purposive sampling to obtain data from at least one representative amongst the various stakeholders, to achieve a sample size of 10-15.

Analyses

The research objectives of the Pathways Study which aim to build theory, and the mixed methods approach to data collection, make realist evaluation an appropriate scientific approach for analysis and interpretation.

A refined middle-range programme theory (Box 1) and subsequent recommendations for practice will ultimately be co-developed as the main outputs of the Pathways Study. The initial programme theory developed from literature (22, and Box 2) will be refined and expanded through examination and analysis of data collected during the Pathways Study, by seeking out semi-regular patterns (demi-regularities, Box 1) of outcomes that occur within the programme theory and then developing causal explanations for these outcomes in the form of context-mechanism-outcome configurations (CMOCs, Box 1) using retroduction (Box 1).(19, 21) Hence, the initial programme theory derived from the literature will provide a series of causal explanations that can be ‘tested’ (confirmed, refuted or refined) against the primary data collected during the Pathways Study.(20) Where indicated by our interpretations of the primary data, new, expanded or revised CMOCs will be used to refine the initial programme theory for use in neonatal units in Kenya – thus further improving the level of understanding captured in the resultant middle-range programme theory. (Figure 2)

Prior to the application of the realist logic of analysis we will do some additional data analysis, for example thematically analysing the qualitative data and using social network approaches to analyse the quantitative data:

- Qualitative data from the study collected during non-participant observations, in-depth interviews and focus groups, and the stakeholder co-design event will initially be analysed thematically using NVivo software. This will lead to inductively

For peer review only - http://bmjopen.bmj.com/site/about/guidelines.xhtml

developing a theoretical framework. Qualitative data on socio-cognitive aspects of the study (key informant interviews and in-depth interviews) will be iteratively analysed using a grounded theory approach following an Open-coding →Axial-coding →Selective-coding approach to develop an explanatory theoretical framework on the role of socio-cognitive skills in the utilization of clinical competencies by nurses.

- Quantitative data collected from a Social Network Questionnaire will be analysed with standard SNA metrics (e.g. density, centrality, etc.) using Gephi free software and/or R to develop sociograms and a set of network measures.

Following the initial phase of realist anlysis and theory building (phase 1, Figure 1), a group of stakeholders will be invited to review and further refine the theory, based on their expert knowledge and experiences (phase 2, Figure 1). Through engagement with these stakeholders, explanatory theory will be further refined, and used to develop a set of recommendations for practice.

Patient and Public Involvement

The Pathways Study research team includes a representative from each of the two hospital study sites and training institutions as study collaborators. The role of study site collaborators is to provide useful linkages for facility engagements before, during and after the study as well as facilitate the smooth conduct of the research by contributing to data collection through arranging for meetings with study participants. During the study, an advisory steering group will be formed of relevant co-investigators who are not directly involved in data collection or ongoing analysis as well as extended colleagues working on relevant projects, to review study findings and provide useful comments and expert advice on study data analysis procedures and interpretation of the study findings.

Ethics and dissemination

For peer review only - http://bmjopen.bmj.com/site/about/guidelines.xhtml

Ethical approval for the Pathways Study has been received from Kenya Medical Research Institute (KEMRI/SERU/CGMR-C/241/4374) and Oxford Tropical Research Ethics Committee (OxTREC 519-22).

Insights from the Pathways Study will add new understanding and a different theoretical lens to ongoing work by KEMRI-Wellcome Trust Research Programme in Kenya, particularly work focused on health workforce communication in neonatal care. Findings will also provide a useful addition to the existing body of literature on social networks of the hospital workforce, which is almost entirely derived from high income settings. The explanatory power of realist programme theory in specifically developing new understanding around the chains of causation associated with social ties of hospital staff, will be useful for designing and targeting interventions to enhance how such ties might positively contribute to change efforts in the neonatal unit. In addition, social network analysis (SNA) is a relatively new methodology in healthcare research, and so the practical learning gained during the research process will be shared with KEMRI-Wellcome Trust Research Programme colleagues and others and will be useful for those intending to use SNA in future research.

Findings will further open-up the window of socio-cognitive skills to policymakers and other stakeholders in Kenya, their role in service delivery quality and by way of interventions influence health workforce socio-cognitive skills strengthening agendas. Findings relating to the role of non-technical socio-cognitive skills in the use of learned clinical competencies in neonatal care, will be used to develop recommendations for nurse educational interventions. Recommendations are likely to include how and when these skills can be incorporated into basic and specialised nursing programmes and in-service continuous professional development training activities. We anticipate that these findings might also be applicable to wider healthcare training programmes.

Findings will be shared with the two study hospitals, relevant educational institutions, and KEMRI-WELLCOME Trust Research Programme and the University of Oxford. Study

For peer review only - http://bmjopen.bmj.com/site/about/guidelines.xhtml

findings will also be disseminated in seminars, local and international conferences, and as academic theses and research articles published in open access scientific journals.

For peer review only

Authors' contributions

This protocol was co-developed by Claire Blacklock (CB) and Conrad Wanyama (CW) as joint authors/co-principal investigators. Mike English (ME), Peris Musitia (PM), Mwanamvua Boga (MB), Geoff Wong (GW), Lisa Hinton (LH) contributed to the development of the protocol, and Juliet Jepkosgei (JJ), Sassy Molyneux (SM) and Jacob McKnight (JM) contributed to the refining of the protocol and study tools for implementation.

Funding statement

The Pathways Study is supported by the Wellcome Trust (#207522) through an award to ME as a Senior Fellowship.

CB is studying for a DPhil - this work is supported by the Nuffield Department of Medicine, University of Oxford and the Medical Research Council [grant number MR/N013468/1].

The funders had no role in the preparation of this report or the decision to submit for publication.

Open Access Policy

This research is funded in whole or in part by the Wellcome Trust (#207522). For the purpose of Open Access, the author has applied a CC-BY public copyright licence to any author accepted manuscript version arising from this submission

Competing interests statement

All authors declare no competing or conflict of interests.

References

1. United Nations Inter-Agency Group for Child Mortality Estimation (UN IGME). Levels & trends in child mortality. Report 2019. New York: UNICEF, 2019. Available at https://childmortality.org/wp- content/uploads/2019/09/UN-IGME-Child-Mortality-Report-2019.pdf

2. Hug L, Alexander M, You D, Alkema L. National, regional, and global levels and trends in neonatal mortality between 1990 and 2017, with scenario-based projections to 2030: a systematic analysis. Lancet Glob Heal. 2019;7(6):e710–20.

3. Keene CM, Aluvaala J, Murphy GAV, Abuya N, Gathara D, English M. Developing recommendations for neonatal inpatient care service categories: Reflections from the research, policy and practice interface in Kenya. BMJ Glob Heal. 2019;4(2).

4. Maina M, Aluvaala J, Mwaniki P, Tosas-Auguet O, Mutinda C, Maina B, et al. Using a common data platform to facilitate audit and feedback on the quality of hospital care provided to sick newborns in Kenya. BMJ Glob Heal. 2018;3(5):e001027.

5. NEST360 (2022 online). Delivering and Sustaining Newborn Technologies. Available at https://nest360.org/technology/

6. Sheikh K, Gilson L, Agyepong IA, Hanson K, Ssengooba F, Bennett S. Building the field of health policy and systems research: Framing the questions. PLoS Med. 2011;8(8):1–6.

7. Scott J, Revera Morales D, Mcritchie A, Riviello R, Smink D, Yule S. Non-technical skills and health care provision in low- and middle-income countries: A systematic review. Med Educ. 2016;50(4):441–55.

8. Lewin S, Reeves S. Enacting “team” and “teamwork”: Using Goffman’s theory of impression management to illuminate interprofessional practice on hospital wards. Soc Sci Med. 2011;72(10):1595–602.

For peer review only - http://bmjopen.bmj.com/site/about/guidelines.xhtml

9. Scott, J. Social network analysis. 2nd ed. Los Angeles: SAGE Publications Ltd, 2000.

10. Rogers EM. Diffusion of Innovations. New York: Free Press; 2003.

11. De Herdt T, de Sardan J. Real governance and practical norms in sub-Saharan Africa. Routledge; 2015.

12. Liberati EG, Tarrant C, Willars J, Draycott T, Winter C, Chew S, et al. How to be a very safe maternity unit: An ethnographic study. Soc Sci Med.. 2019;223(January):64–72.

13. Liberati EG, Tarrant C, Willars J, Draycott T, Winter C, Kuberska K, et al. Seven features of safety in maternity units: a framework based on multisite ethnography and stakeholder consultation. BMJ Qual Saf. 2020;bmjqs-2020-010988.

14. Prineas S, Mosier K, Mirko C, Guicciardi S. Non-technical Skills in Healthcare BT - Textbook of Patient Safety and Clinical Risk Management. In: Donaldson L, Ricciardi W, Sheridan S, Tartaglia R, editors. Cham: Springer International Publishing; 2021. p. 413–34.

15. Coleman JS, Katz E, Menzel H. Medical innovation: A diffusion study. Indianapolis: Bobbs-Merrill Company; 1966.

16. Donohue JM, Guclu H, Gellad WF, Chang CC, Huskamp HA, Choudhry NK, Zhang R, Lo-Ciganic WH, Junker SP, Anderson T, Richards-Shubik S. Influence of peer networks on physician adoption of new drugs. PloS one. 2018 Oct 1;13(10):e0204826.

17. Evan Pollack C, Wang H, Bekelman JE, Weissman G, Epstein AJ, Liao K, et al. Physician Social Networks and Variation in Rates of Complications after Radical Prostatectomy. Value in Health. 2014;17(5):611–8. Available from: http://dx.doi.org/10.1016/j.jval.2014.04.011

18. Dixon-Woods M, Bosk CL, Aveling EL, Goeschel CA, Pronovost PJ. Explaining Michigan: developing an ex post theory of a quality improvement program. Milbank Q. 2011 Jun;89(2):167-205. doi: 10.1111/j.1468-0009.2011.00625.x. PMID: 21676020; PMCID: PMC3142336.

19. Wong G, Westhorp G, Pawson R, Greenhalgh T. Realist Synthesis. RAMESES Training Materials. RAMESES Proj. 2013;(July 2013):55.

For peer review only

20. Pawson, R, Tilley, N. Realistic evaluation. London: SAGE Publications Ltd, 1997

21. Greenhalgh T, Pawson R, Wong G, Westhorp G, Greenhalgh J, Manzano A, Jagosh J. Retroduction in realist evaluation. The RAMESES II Project. 2017.

22. Blacklock C, Darwin A, English M, McKnight J, Hinton L, Harris E, Wong G. The social networks of hospital staff: a realist synthesis. J Health Serv Res Policy. 2022;0(0):1–11.

23. Wong G, Westhorp G, Manzano A, Greenhalgh J, Jagosh J, Greenhalgh T. RAMESES II reporting standards for realist evaluations. BMC Med. 2016;14(1):1–18.

24. Tuti T, Bitok M, Malla L, Paton C, Muinga N, Gathara D, et al. Improving documentation of clinical care within a clinical information network (CIN): An essential initial step in efforts to understand and improve care in Kenyan hospitals. BMJ Glob Heal. 2016;1(1):1–8.

For peer review only

For peer review only - http://bmjopen.bmj.com/site/about/guidelines.xhtml

Figure legend

Figure 1: A summary of mixed methods used in the Pathways Study

The figure describes the methodological approach for data collection in phase 1 (in the first three horizontal boxes), which involve data collection from the two case study hospitals and additional data from a nursing interest group, that will comprise nurses providing neonatal nursing care in the two case study hospitals and possibly other hospitals within Kenya. During this phase 1 work, we will also form a technical group for defining a realist evaluation programme theory. The last box, below the 3 boxes, describes a phase 2 data collection, where study findings from the phase 1 study participants as well as the initially developed programme theory, will be discussed over a one-day workshop by a group of prior nominated stakeholders. These stakeholders will be drawn from memberships of neonatal nurses, neonatal and paediatric consultants, neonatal and paediatric professional associations, Kenya's Ministry of Health, division of newborn care, Kenya's medical and nursing practice regulatory agencies and senior health systems researchers from KEMRI-Wellcome Trust Research Programme. It is the recommendations from these stakeholder group that will inform the design of interventions targeting behaviour change among health workers providing care to neonates in Kenya.

Figure 2: Programme Theory Development in the Pathways Study

The figure shows the dynamic and iterative process of theory development and refinement in the Pathways Study, starting from the initial programme theory, to the development of middle-range programme theory and recommendations for practice, using study data, steering group and stakeholder inputs, continual researcher collaboration and relevant literature.

Study tools: Appendix A-F attached as supplementary material

For peer review only - http://bmjopen.bmj.com/site/about/guidelines.xhtml

Figure 1: Summary of mixed methods used in the Pathways Study

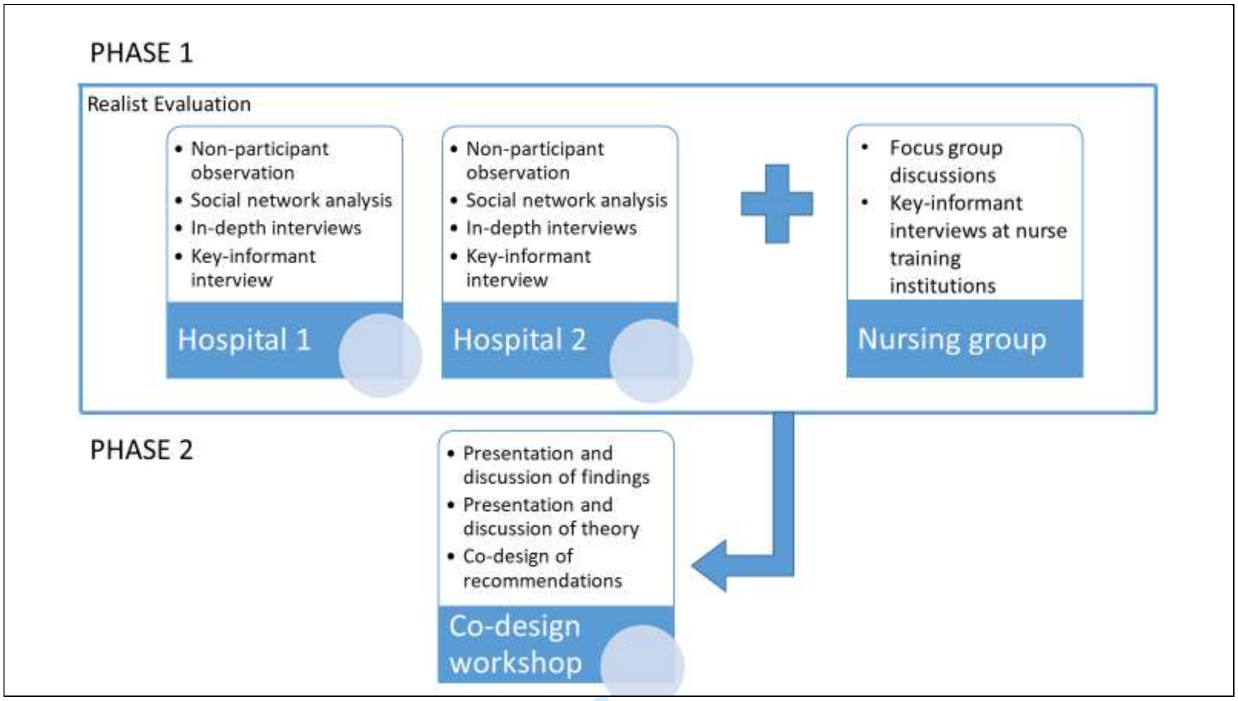

Figure 2: Programme theory development in the Pathways Study

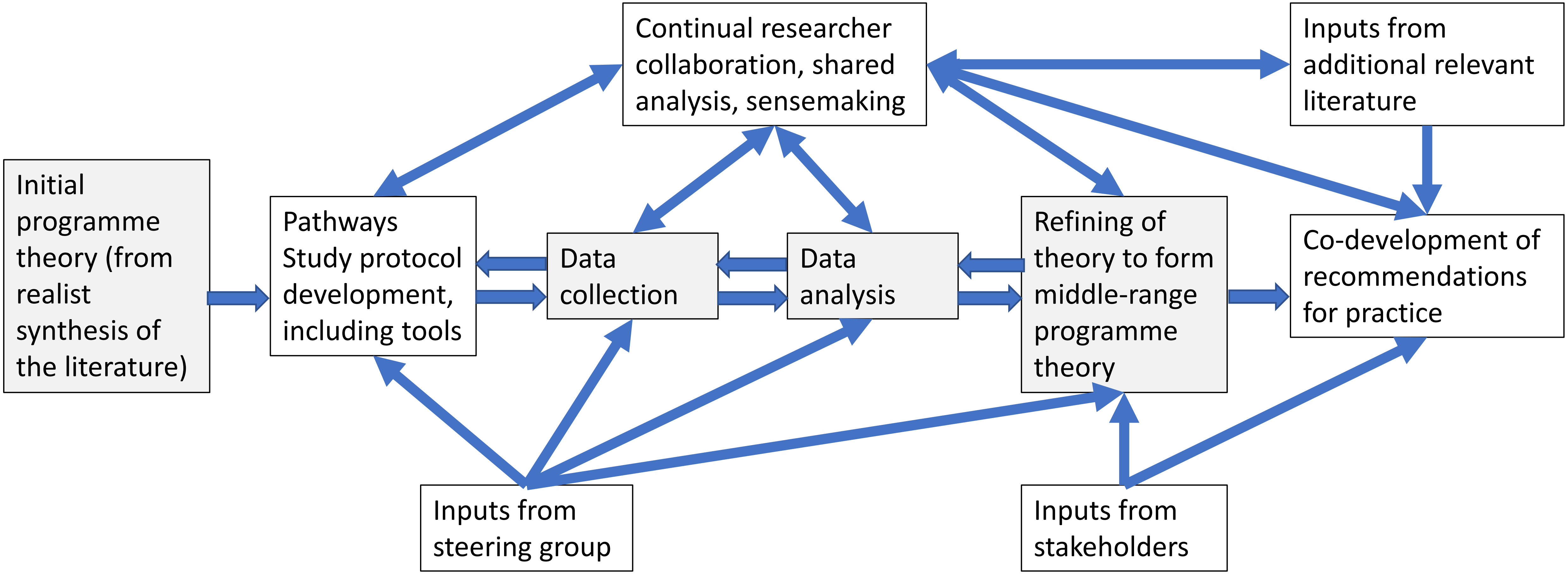

Note: Data collection and data analysis will be undertaken in an ongoing manner, so that findings can inform study tools to further confirm, refute or refine tentative emerging causal explanations.

Appendix A: Pathways Study: Non-participant observation prompt list

Before you start:

Introduce yourself to the staff you will be observing, explain the study, and obtain verbal consent (**record verbal consent on the relevant form**).

Note down:

- Date, time, duration, and location of non-participant observation
- Which cadres of staff/students you observe
- Numbers of staff you observe

Level A: Administration, Leadership and Management

1. **Where is the Administration block located in relation to the health care service delivery units?** Which offices and posts are located in the administration block?
2. **Is there a notice board?** What is posted on the notices?
3. **Is there a service charter?** What is contained on the charter? Where is it displayed?
4. **Is there a company/institutional mission, vision, values displayed?** Describe this.
5. **Where is the CEO's office OR medical superintendent's office in relation to other offices?** What is contained in the office: Furniture, artefacts, symbols, wall hangings?
6. **What is contained in the head of nursing's office?** Furniture, artefacts, symbols, wall hangings?
7. **What is the nature of dressing for the hospital top management?** What is the colour? Do they have the facility logo?
8. **What is the nature of dressing for hospital staff?** Does it differ from that of top management? Do they have facility logo? Are they uniforms? Are there dressings with no logos or with any other logos?
9. **How is the parking organised?** Management versus staff
10. **Which meetings were observed?** What was the purpose of the meeting? Was the meeting by formal invitation (any evidence to this?) Where were they held? Who was chair? How are people seated? Who spoke to whom? Any commonly used words/phrases? Any common verbal and non-verbal cues? **Are there any meetings before/after the main meeting?** How are the members constituted? How are they conducted? How long do they last? What happens thereafter?

Level B: Units/Wards

1. **Who are staff generally communicating with?** Within their own cadre/seniority level? Between different cadres/seniority levels?
2. **Do they communicate with different colleagues depending on what they are communicating about?** Which colleagues to staff appear to communicate freely with? Are there any barriers between certain staff? What is the style of communication of different staff?
3. **Do all staff appear to be equally free to communicate with their colleagues?** If not, who is not able to?
4. **What appear to be the norms of staff behaviour in the location you are observing?** Do some cadres of staff appear to have different behavioural norms to others?
5. **Do staff appear to follow advice of their colleagues in their work/decision-making?** Can you describe?
6. **Which staff seem to be more influential in the location you are observing?** What made you form this assessment/why?
7. **Which staff seem less influential in the location you are observing?** What made you form this assessment/why?
8. **Can you notice a hierarchy in the location you are observing?** Please describe.
9. **Do staff use any other information sources to undertake their work?** Books, protocols, etc?
10. **Can you observe any mentoring/supervising/supporting relationships or behaviours between staff?** How formal/informal is it? How is the relationship between the mentor and mentee(s)?
11. **Any unit-level meetings observed?** Describe Type, purpose of the meeting, where the meeting is held, who attended the meeting, who chairs the meeting, how are people seated? Who speaks to who? Any words or phrases that are commonly used? Any common verbal and non-verbal cues? **Are there any meetings before/after the main meeting?** How long do these last? How are the membership informally constituted? How are they are conducted? What happens thereafter?

For peer review only

Appendix B: Pathways Study - In-Depth Interview Guide

(Informed consent must be provided by the participant before commencing the interview.) *My name is X and I am a researcher working on the Pathways Study. I am interested in learning more about how nursing graduates (staff) who work in neonatal care utilise knowledge and skills learnt in nursing programmes, and how this is shaped their relationships at work and their peers and other clinical staff. I would like to ask you a few questions.*

Participant Study ID number:

PART A – for all participants

1. **How do you decide/choose who to ask for help or information at work?** Can you give me an example? Why did you choose this person? What was it about them/you/the situation, which made you ask them? Does choice of colleague depend on the type of help or information you need? Which colleagues do you need to speak to on a day-to-day basis to undertake your role effectively? Why?
2. **Does discussion with a colleague at work change what you do/ your behaviour/ your decision making?** Can you give me an example?
3. **Whose opinion(s) do you value most at work?** Why? Anything about them/you/the situation?
4. **Are there any colleagues that you find difficult to speak to (ask for help from) at work?** Why do you think you find it difficult to speak to (ask for help from) them? Anything about them/you/the situation? Are there any situations when you would speak to (ask for help from) them? Do you feel comfortable approaching senior (or junior) colleagues for help? Do you feel comfortable approaching colleagues from different professions for help?
5. **How do you select with whom to share your helpful information or knowledge (i.e. information that you have)?** What kinds of information or knowledge do you share?
6. **Are there any colleagues with whom you find it difficult to share your helpful information or knowledge?**
7. **Are there situations where you have to ask for information or help but you cannot get it?** Can you give me an example: situation/type of help/from who? What did you do about it: yourself/situation/the other person(s)? How does this make you feel?
8. **Do you feel staff members share information and knowledge freely at your hospital/unit?** Can you give me an example? What type of information, knowledge or help would people be commonly seek from others, or share with others on a day-to-day basis? Why do you think this is so?
9. **What would help you better communicate with your colleagues at work?** Would this improve patient care? How? Why?
10. **Can you always access the information you need in the workplace?** Do you ever feel like you are missing out on information at work? Do you try to access the information in any other ways? For example: clinical guidelines, protocols, hospital policies, key communication messages from management to staff, information specific to the professional cadre, books, phone, WhatsApp groups, any other information?
11. **Do you use any other information sources/resources/courses/CPD to do your job?** Can you give an example? How and when do you access this information?
12. **How could access to essential and reliable information be improved in the unit/hospital?** Would this improve patient care? How? Why?
13. **Do you feel that you can always say and do what you think is best, in the workplace?** Why/why not? Please give me an example. Do you feel like you do things differently or have a different outlook/attitude to some of your colleagues? How does this make you feel? Please give me an example. How does this make your colleagues feel? Does this impact on your patient care?
14. **What are your experiences of supervision/support/mentoring at work?**
15. **What (or who) do you feel has influenced you the most in the way you work?** Can you describe this type of influence detail? How has this impacted your individual perception about your work? How has this impacted on how you provide care to your patients? How does this make you and colleagues feel?
16. **Do you feel there is anyone at work who influences the behaviour of your whole peer group?** Who? Why do you think this is? Do you think you contribute to this person influencing your peer group? How?
17. **Are there individuals you prefer to be on duty with?** Who? What is it about them that makes you want to only work with? What is it about you that makes you wish to work with them?
18. **Are there individuals you would prefer not to be on duty with?** Who? What is it about them that makes you wish not to work with them? What is it about you that makes you wish not to work with them?
19. **What do you do if you receive contradictory advice at work?** Where does the contradictory advice come from? Talk me through your thought processes in how you decide what you will do.
20. **What are the hospital's 'values'?** How are these expressed to you? Do these 'values' change what you do at work?

For peer review only

21. **Can you describe what is known and said about this hospital by the general public?** Do you think this perception is also shared by some (or most) staff members? What do you think has contributed to this? How has this influenced the way you and your colleagues provide care to your patients?

For non-nurse staff and students, interview ends here.

PART B: additional questions for nurse managers, all nurses, plus student nurses

20. **What is your experience with new graduate nurses in providing care to neonates?** What makes you say so?
21. **Is there supervision/support/mentorship at workplace?** To whom is it targeted? Who implements it? When does this happen? What has been your experiences of supervision/support/mentoring at work? Can you give me an example? (nurses and student nurses)
22. **Does the workplace plan and implement off-the job formal training (including both short and long training opportunities) aimed at improving patient care at work?** Can you give an example? **If you have attended any of these trainings, c**an you comment on your experience? Do you feel you have utilised what you learned? What makes you say so? Are there any specific workplace factors that contributed to this? What are they? (nurses)
23. **What stands out as a factor(s) that determines how nurses are likely (or not) going to utilise that they have learned at their workplace?** Tell me about institutional leadership factors, mentorship factors, other institutional initiatives (which, for who, how, when)? (nurses and student nurses)
24. **Tell me about individual nurse-specific factors that you have observed that determine how likely nurses are to use what they have learned in their workplace** (looking for things like effective communication, teamwork self-awareness, decision making etc----human factors)? (nurses and student nurses)
25. **Did you feel you are confident in communicating with rude, angry or disrespectful patients/colleagues?** Why do you say so? Can you give me an example?
26. **Are there emotionally challenging situations that you find handling at workplace?** Give me an example? Why do you say so? How does this impact on the care you give to your patients?
27. **Do you think there are factors about your other professional colleagues (doctors, nutritionists, laboratory personnel etc) that contribute to how nurses utilize knowledge and skills and competencies acquired during their training?** What are these factors? Why do you say so? (nurses and student nurses)
28. **Do you think the nursing school training theoretical work (including in clinical skills labs) is aligned to what is expected at the workplace?** Can you comment on this as a student nurse, a newly qualified nurse and as an experienced manager/leader?

For peer review only

Appendix C: Pathways Study – Social Networks Questionnaire

Informed consent must be provided by the participant before commencing the SNA questionnaire interview.

My name is X and I am a researcher working on the Pathways Study. We are interested in learning more about how staff/students who work in neonatal care communicate with one another, and how this might influence what they do at work. I would like to ask you a few questions about your own experiences of communicating with your colleagues.

Demographics

- Study ID number
- Job title/cadre/student status (include qualification and level of seniority)
- Unit (maternity, postnatal, neonatal, paediatric)
- Year of birth (or age in years)
- Gender
- Highest qualification (and year)
- Specialist certificates
- Training courses/workshops attended (in the last 5 years)
- Involvement in research
- Academic/teaching/faculty position
- Nomination as a clinical mentor/champion/coach/preceptor
- Length of time working on ward
- Employment elsewhere

Scenarios (will be piloted and adapted at start of study)

1. You are not sure of the dose of medication to give to a neonate.
   List #1: Please indicate if you would speak to/seek advice from anyone

2. A neonate has an infection and you are unsure of the treatment plan.
   List #1: Please indicate if you would speak to/seek advice from anyone

3. You need help to perform a routine procedure on a stable neonate.
   List #1: Please indicate if you would speak to/seek advice from anyone

4. You have a patient on the ward with clinical orders that you are sure do not comply with clinical practice guidelines.
   List #1: Please indicate if you would speak to/seek advice from anyone

5. You have a patient on the ward who you think needs a certain specific care, but it is not available.
   List #1: Please indicate if you would speak to/seek advice from anyone

6. You notice that a neonate is looking very sick.
   List #1: Please indicate if you would speak to/seek advice from anyone

7. You have had a very busy shift and two neonates died today. You are feeling tired and upset.
   List #1: Please indicate if you would speak to/seek advice from anyone

8. The unit has no water.
   List #1: Please indicate if you would speak to/seek advice from anyone

9. A colleague has been rude to you.
   List #1: Please indicate if you would speak to/seek advice from anyone

10. You are very busy and some of your colleagues have finished their tasks.
    List #1: Please indicate which colleagues would likely offer to help you with your own tasks

11. A mother has refused a procedure for her baby.
    List #1: Please indicate if you would speak to/seek advice from anyone

12. A baby has died on the unit and you are uncomfortable breaking the bad news to her.

For peer review only

List #1: Please indicate if you would speak to/seek advice from anyone
List #1 = Roster method – a list of staff working in or supporting the unit

For peer review only

Appendix D1: Pathways Study – Key informant Interview

for Hospital Nursing Team Leader/Manager (one per hospital/unit)

(Informed consent must be provided by the participant before commencing the interview.) *My name is X and I am a researcher working on the Pathways Study. I am interested in learning more about how nursing graduates (staff) who work in neonatal care utilise knowledge and skills learnt in nursing programmes, and how this is shaped their relationships at work and their peers and other clinical staff. I would like to ask you a few questions.*

Participant study ID number:

Interview schedule: (Schedule will be piloted and adapted at start of study. Questions will also be adapted for interviewing researchers, as per protocol)

1. **Can you describe your role in unit** (Shift leader, unit manager etc).
2. **Does your unit/hospital have any job/role descriptions for nurses working on the unit?** Please can I see a copy?
3. **Does your unit/hospital have any Continuing Professional Development policies?** Please can I see a copy? Does the hospital have a library? Any annual CPD requirements for staff? Appraisals? Etc.
4. **Does your unit/hospital have any regular clinical meetings?** Who attends? What is the format? How frequently? Etc.
5. **Are any on-the-job training schemes going on in your unit/hospital for nurses?** Courses? Mentoring? Etc.
6. **What is your total nursing staff?** What is your current patient: nurse ratio? What is your expected ration?
7. **Do you have a nursing staff rota for each shift**? Can I have a copy of the last week and this week?
8. **How many nurses are you likely to be having on each shift?** AM, PM, Night, Weekends? What is the typical composition of this rota? Why is that so? Is this defined by the institution? Any other factors (e.g NCK etc?) (Nurses and student nurses)

For peer review only

Appendix D2: Pathways Study – Key informant Interview

for Nurse educators with administration and programme management role (one per teaching institution)

(Informed consent must be provided by the participant before commencing the interview.) *My name is X and I am a researcher working on the Pathways Study. I am interested in learning more about how nursing graduates (staff) who work in neonatal care utilise knowledge and skills learnt in nursing programmes, and how this is shaped their relationships at work and their peers and other clinical staff. I would like to ask you a few questions.*

Participant study ID number:

Interview schedule: (Schedule will be piloted and adapted at start of study. Questions will also be adapted for interviewing researchers, as per protocol)

9. **What is your role in the college** (Lecturer/tutor, programme manager, head of department etc).
10. **How many nursing programmes do you have in the school?** What is the level of training for each? What is the course duration of each?
11. **Do you have a curriculum for your nursing programmes?** Can I have a copy please? **What is your curricular review/revision policy/plan?** Can I have a look at it please?
12. **Are the nursing programmes all accredited?** Who is the accreditor? Can I see evidence of accreditation please?
13. **What is the entry criteria for each?** How do you determine the entry criteria? Do you have evidence for this?
14. **What is the admission capacity for each programme?** How is this determined?
15. **How many nurses from the programmes graduate annually?** Do you have any dropouts?
16. **Where do your nursing students undertake their clinical practicum?** Do you have any formal agreements with these institutions? Can I see the evidence please?
17. **Is there any mutual working relationship with facilities that provide student practicum placements in the area of nursing staff development trainings (workshops, symposiums, library, clinical consultancy)?** If you have evidence for this, can I see it please?
18. **What is your total academic staff (includes clinical laboratory and clinical instructors)?** What is your current Student: Faculty ratio? What is your expected ratio?

For peer review only

Appendix E1 - Pathways Study - FGD: Nursing educators (Teaching faculty involved in the curriculum implementation of nurse trainings for both basic and specialist neonatal and/or paediatric nurses, 5-9 members per FGD)

(Informed consent must be provided by each of the participant before commencing the discussion.) *My name is X and I am a researcher working on the Pathways Study. I am interested in learning more about how basic and specialist nursing graduates (staff) who work in neonatal care units are prepared for their workplace and how this preparation influences how they utilise knowledge and skills learnt in their nursing training. I would like us to have a discussion about your own experiences.*

1. Welcome the participants, do a formal introduction and allocate **Participant study ID numbers:**
2. Explain the process and lay ground rules
3. Explain the need to record
4. Gain verbal consent for participation and recording

Main question	Additional questions
What is your experience of how well Kenya's nurse training programmes prepare nurses to work in neonatal care units?	• Start off with basic programmes<br>• Then ask about Specialist programmes<br><br>Ask Why they say so? Give examples
In your experience as educators, what has worked to adequately prepare any nurse, basic or specialist, to be able to easily use what they have learned in college at their workplaces?	Probe on people-related factors<br>Probe on leadership related factors<br>Probe on assertiveness, self-awareness and decision-making factors
In your experience as educators, what has hindered preparing any nurse, basic or specialist, to be able to easily use what they have learned in college at their workplaces?	Probe on people-related factors<br>Probe on leadership related factors<br>Probe on assertiveness, self-awareness and decision-making factors
Do you think the theoretical work (including in clinical skills labs) is aligned to what is expected at the workplace for your future graduate nurses?	Probe on how this is scheduled, delivered and perceived by both teachers and learners
Do you think the communication skills and emotional competence skills taught at the nursing school is aligned to the challenges nurse graduates face at the workplace?	Probe on how these skills are taught and perceived by both teachers and learners
In your experience as educators, what things/factors about nursing students themselves have helped them, basic or specialist, to be able to easily use what they have learned in college at their workplaces?	Probe on people-related factors<br>Probe on leadership related factors<br>Probe on assertiveness, self-awareness and decision-making factors
In your experience as educators, what things/factors about nursing students themselves have hindered them, basic or specialist, to be able to easily use what they have learned in college at their workplaces?	Probe on people-related factors<br>Probe on leadership related factors<br>Probe on assertiveness, self-awareness and decision-making factors
In your experience as educators, what things/factors about hospital staff as clinical preceptors/clinical mentors do you think have helped nurses, basic	Probe on people-related factors<br>Probe on leadership related factors

For peer review only

or specialist, to be able to easily use what they have learned in college at their workplaces?	Probe on assertiveness, self-awareness and decision-making factors
In your experience as educators, what things/factors about hospital staff as clinical preceptors/clinical mentors have hindered nurses, basic or specialist, to be able to easily use what they have learned in college at their workplaces?	Probe on people-related factors<br>Probe on leadership related factors<br>Probe on assertiveness, self-awareness and decision-making factors
Do you think there are factors about your other professional colleagues (doctors, nutritionists, laboratory personnel etc) that contribute to how nurses utilize knowledge and skills and competencies acquired during training?	Probe to identify these factors (probe on teamwork, team leadership, decision making and other human factor development through collaborative social learning)<br><br>Why do you say so?
What has been the role of the teaching institution (any initiatives) in responding to the challenges you have mentioned above: challenges at<br>• Teaching faculty level<br>• Students' level<br>• Hospital staff level	Probe further on these initiatives, what, when, how, for whom?
Are you involved in these initiatives?	Probe further on this: What, as who, when, how, for whom?
Do you have any recommendations going forward?	
Is there anything else from anyone before we close?	
Thank the participants for their time and contributions	

For peer review only

Appendix E2 - Pathways Study - FGDs: 1) Nursing graduates **(Basic Diploma and Degree General Nursing graduates)** working in various units and providing caring for newborn babies, and **2) Specialist Nursing graduates (Specialist Neonatal and/or Paediatric Nurses and/or maternal-neonatal nurses)** working in various units and providing caring for newborn babies (5-9 members per FGD).

(Informed consent must be provided by each of the participant before commencing the discussion.) *My name is X and I am a researcher working on the Pathways Study. I am interested in learning more about how nursing graduates (staff) who work in neonatal care utilise knowledge and skills learnt in nursing programmes or other neonatal short CPD programmes. I would like us to have a discussion about your own experiences.*

5. Welcome the participants, do a formal introduction and allocate **Participant study ID numbers:**
6. Explain the process and lay ground rules
7. Explain the need to record
8. Gain verbal consent for participation and recording

Main question	Additional questions
Can you describe to me the nature of work you are involved in in this unit/department?	Are there any challenges you have experienced in undertaking your role? Give examples and why?
For Basic Diploma/Degree General Nurses: How would you describe your basic training in preparing you to work in neonatal care units?<br><br>*For Specialist Neonatal and/or Paediatric Nurses and/or Maternal-neonatal Nurses:* How would you describe your specialist training in preparing you to work in neonatal care units? How does this compare with your basic nursing training?	Why do you say so? Give examples<br><br>Probe on preparedness on communication skills and handling emotions (ability to communicate well with patients, colleagues, and supervisors. Handling angry parents, breaking bad news & handling rude/ disrespectful colleagues)<br>Recognizing and managing own stress
Have you undertaken any neonatal-training relevant short course/training? Describe it: what, when, content, how and relevance	How does it compare to your basic nursing training? Why do you say so? Give examples.
What things/factors about yourself have helped you use what you learned in college and/or in short courses?	Probe on people-related factors<br>Probe on leadership related factors<br>Probe on assertiveness, self-awareness and decision-making factors
What things/factors about working with others have helped you use what you learned in college and/or in short trainings?	Probe on people-related factors<br>Probe on leadership related factors<br>Probe on assertiveness, self-awareness and decision-making factors
What things/factors about yourself have not helped you use what you learned in college and/or short trainings?	Give examples. Probe as above. Are these factors shared by others? What do the rest of us think?

For peer review only

What things/factors about working with others have hindered your ability to use what you learned in college and/or short trainings?	Give examples. Probe as above. Are these factors shared by others? What do the rest of us think?
Do you think your colleagues elsewhere share your feelings? (ask about Private hospitals versus public hospitals)	Why do you think this is so?
What is your opinion about practice improvement initiatives such as?<br>1. CPD courses (give examples)<br>2. Workplace mentorship<br>3. Using clinical practice guidelines<br>4. Staff induction trainings<br>5. Staff appraisal and performance management	Probe on How it is done, Why it is done, for Whom it is done, When it is done
If you are involved in any of the mentioned practice improvement initiatives above (need to say which one), share your experiences.	Ask: whether the experience is as a recipient or as a provider. Ask them to describe: How, When and Why.
Do you have any recommendations about this?	Ask about:<br>1. Recommendations for nurses as practitioners<br>2. Recommendations about other clinical colleagues<br>3. Recommendations for hospital managers<br>4. Recommendations for Nursing schools<br>5. Recommendations for the regulator
Is there anything else from anyone before we close?	
Thank the participants for their time and contributions	

For peer review only

Appendix F - Pathways Study – Stakeholder workshop event schedule (10-15 members)

Event programme/ timetable (if to be held online instead of in-person, the programme will be adapted accordingly)

Time	Activity	Description
10am	Consent<br>Registration and refreshments	Invited stakeholders will be asked to give consent before participating in the event. They will have previously received a participant information sheet by email and will be given the opportunity to ask any questions in person, before completing the consent form.<br>Reimbursements will be given to participants.
10.30am	Welcome	Participants will be welcomed to the event by the research team.
10.45am	Introduction to study	Researchers will present the background of the study, including findings of a realist synthesis, and work conducted on the neonatal unit in Kenya.
11.15am	Group brainstorming session – how do social ties influence quality of patient care?	Participants will be asked to discuss their initial thoughts on the information/issues in the presentation and how this compares to personal experiences, in small groups. Groups will be provided with paper, sticky notes, and flip chart.
11.45am	Feedback to whole group	Each small group will be encouraged to share their thoughts and take any questions.
12.15pm	Lunch	
1pm	Presentation of findings from interviews, SNA and non-participant observation	Researchers will present the remaining findings of the work conducted on the neonatal unit in Kenya to the stakeholders. This will include a series of summary slides and handouts which the participants can use in the activity.
1.30pm	Group activity – what do we think of the findings? how should these findings be used?	Participants will be asked to discuss the findings and put down their thoughts on paper, to be able to present back to the whole group.
2pm	Feedback to whole group	Each small group will be encouraged to share their thoughts and take any questions.
2.30pm	Tea	
3pm	Group discussion – what should our recommendations be?	During the tea break, the researchers will have collated the main points from the previous discussions and summarised into themes. These themes will be used as prompts for discussion.<br>The session will be flexible, adapting based on the dynamics of previous sessions but is likely to involved facilitated group work, facilitated group discussions, and ranking exercises or similar.
4.30pm	Summary and close	Stakeholders will be thanked for their participation and given information about how findings will be fed back to them and others.

For peer review only
